# Improving the drug-likeness of inspiring natural products - evaluation of the antiparasitic activity against *Trypanosoma cruzi* through semi-synthetic and simplified analogues of licarin A

**DOI:** 10.1038/s41598-020-62352-w

**Published:** 2020-03-25

**Authors:** Thiago R. Morais, Geanne A. Alves Conserva, Marina T. Varela, Thais A. Costa-Silva, Fernanda Thevenard, Vitor Ponci, Ana Fortuna, Amílcar C. Falcão, Andre G. Tempone, João Paulo S. Fernandes, João Henrique G. Lago

**Affiliations:** 1 Institute of Environmental, Chemical and Pharmaceutical Sciences, Universidade Federal de São Paulo, São Paulo, 09972-270 Brazil; 2 Center of Natural Sciences and Humanities, Universidade Federal do ABC, São Paulo, 09210-580 Brazil; 3 Laboratory of Pharmacology, Faculty of Pharmacy of University of Coimbra, 3000-370 Coimbra, Portugal; 4 CIBIT/ICNAS – Coimbra Institute for Biomedical Imaging and Translational Research, University of Coimbra, 3000-370 Coimbra, Portugal; 5 Centre for Parasitology and Mycology, Instituto Adolfo Lutz, São Paulo, 01246-000 Brazil

**Keywords:** Drug discovery and development, Organic chemistry, Natural product synthesis

## Abstract

Neolignan licarin A (**1**) was isolated from leaves of *Nectandra oppositifolia* (Lauraceae) and displayed activity against trypomastigote forms of the etiologic agent of American trypanosomiasis, *Trypanosoma cruzi*. Aiming for the establishment of SAR, five different compounds (**1a – 1e**) were prepared and tested against *T. cruzi*. The 2-allyl derivative of licarin A (**1d**) exhibited higher activity against trypomastigotes of *T. cruzi* (IC_50_ = 5.0 μM and SI = 9.0), while its heterocyclic derivative **1e** displayed IC_50_ of 10.5 μM and reduced toxicity against NCTC cells (SI > 19.0). However, these compounds presented limited oral bioavailability estimation (<85%, Papp <1.0 × 10^−6^ cm/s) in parallel artificial membrane permeability assays (PAMPA) due to excessive lipophilicity. Based on these results, different simplified structures of licarin A were designed: vanillin (**2**), vanillyl alcohol (**3**), isoeugenol (**4**), and eugenol (**5**), as well as its corresponding methyl (**a**), acetyl (**b**), O-allyl (**c**), and C-allyl (**d**) analogues. Vanillin (**2**) and its acetyl derivative (**2b**) displayed expressive activity against intracellular amastigotes of *T. cruzi* with IC_50_ values of 5.5 and 5.6 μM, respectively, and reduced toxicity against NCTC cells (CC_50_ > 200 μM). In addition, these simplified analogues showed a better permeability profile (Papp > 1.0 × 10^−6^ cm/s) on PAMPA models, resulting in improved drug-likeness. Vanillyl alcohol acetyl derivative (**3b**) and isoeugenol methyl derivative (**4a**) displayed activity against the extracellular forms of *T. cruzi* (trypomastigotes) with IC_50_ values of 5.1 and 8.8 μM respectively. Based on these results, compounds with higher selectivity index against extracellular forms of the parasite (**1d**, **1e**, **3d**, and **4a**) were selected for a mechanism of action study. After a short incubation period (1 h) all compounds increased the reactive oxygen species (ROS) levels of trypomastigotes, suggesting cellular oxidative stress. The ATP levels were increased after two hours of incubation, possibly involving a high energy expenditure of the parasite to control the homeostasis. Except for compound **4a**, all compounds induced hyperpolarization of mitochondrial membrane potential, demonstrating a mitochondrial imbalance. Considering the unique mitochondria apparatus of *T. cruzi* and the lethal alterations induced by structurally based on licarin A, these compounds are interesting hits for future drug discovery studies in Chagas disease.

## Introduction

Chagas disease (CD) is a parasitic infection caused by *Trypanosoma cruzi*, endemic in Latin America and recently found in non-endemic countries of North America, Europe and Asia. The main form of transmission depends on the presence of a vector, being *Triatoma infestans* the most common^1^. It is estimated that over eight million people are infected by the parasite and 70 million are at risk, living on endemic areas or in places where the control of other forms of transmission (oral, blood transfusion or organ transplant) is not rigid^2^. CD is a two-stage disease: the acute phase, characterized by the high parasitaemia, and invasion by trypomastigote forms of different organs and the chronic phase, which may be latent for decades before appearance of clinical signs and usually associated with the development of cardiomyopathy^3,4^. The available therapy for the treatment of CD is actually restricted to two approved drugs: benznidazole and nifurtimox, remains a controversial issue, with contradictory results in the chronic phase of the disease^5–8^. Furthermore, these nitrocompounds showed several side effects associated to prolonged treatment regimens.

Several clinical trials involving drug-repositioning (such as antifungal azoles) were carried out as well as studies involving new molecules, but none have reached the market yet^4^. Therefore, new effective drugs for the treatment of CD are still needed. The *Drugs for Neglected Diseases initiative* (DNDi) defines that a desirable hit compound should present considerable efficacy (IC_50_ < 10 µM), selectivity (>10-fold over mammalian cells) and adequate oral bioavailability^9^.

Natural products have always been a source of a great variety of bioactive molecules, mostly substances from the organism secondary metabolism. Many drugs available in the market are natural products as found in nature or compounds designed based on the structure and activity of these natural products (semi-synthetic or completely synthetic)^10^. The biodiversity of plants makes them a commonly explored source of novel bioactive compounds, providing molecules with distinct structures, complex or simple, with huge chemical variety^11^. Several research groups are focused on the isolation and identification of novel compounds with antimicrobial activity from plant extracts, aiming to use them as prototypes for drug discovery against Chagas disease^12^. In this context, licarin A is a neolignan isolated from different plant species with reported activity against *Mycobacterium tuberculosis*^13,14^, *Schistosoma mansoni*^15^, *Trypanosoma cruzi*^15–17^, and *Leishmania major*^18^. Considering the promising activity against *T. cruzi* and the considerable amounts of licarin A isolated from the leaves of *Nectandra oppositifolia* (Lauraceae), this compound was selected for preparation of semi-synthetic analogues to further pharmacophore exploitation. Thereafter, licarin A was obtained in pure form and five semi-synthetic and twenty-one analogues were designed by the molecular simplification approach. The main objective was to assess a structure-activity relationship (SAR) for the antiparasitic activity of licarin A, determine the pharmacophore of these molecules, to predict their oral bioavailability through the *in vitro* parallel artificial membrane permeability assay (PAMPA), and also to study the mechanism of action of the compounds with higher selectivity.

## Results and Discussion

### Chemical characterization of licarin A (1)

NMR (^1^H and ^13^C) and HRESIMS data of the isolated compound from *n*-hexane extract from leaves of *N. oppositifolia* were compared with those reported in the literature^19^, allowing the identification of licarin A in 99% of purity as indicated by HPLC.

### Design and preparation of licarin A analogues

**Figure 1:**
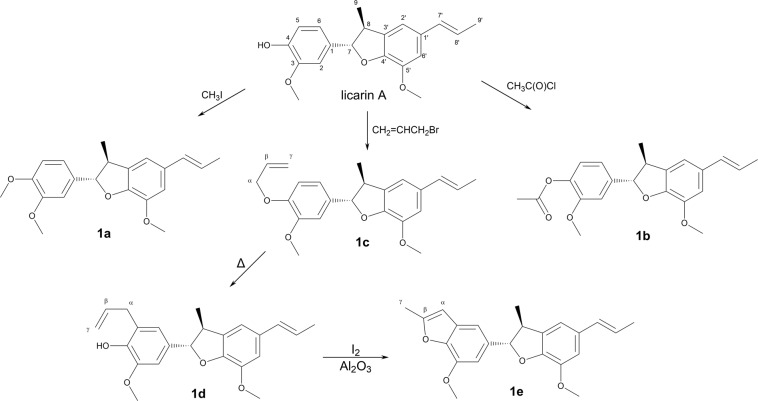
Licarin A (**1**) and semi-synthetic derivatives **1a – 1e**.

**Figure 2:**
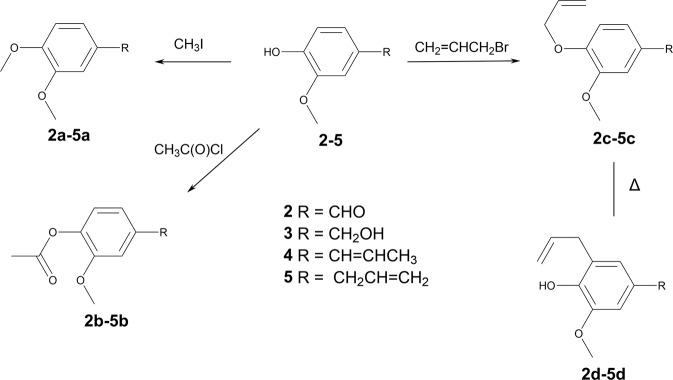
Simplified analogues (**2a-d – 5a-d**) of licarin A (**1**).

Five semi-synthetic analogues of licarin A (compounds **1a**–**1e**, Fig. 1) were prepared through classical organic processes such as methylation, acetylation, allylation and Claisen rearrangement. Compound **1e** was obtained through iodine-promoted cyclization of the corresponding *ortho*-allylphenol followed by deiodination with basic alumina using a previously reported method^20,21^. Their structures were confirmed by analysis of NMR (^1^H and ^13^C) and HRESIMS data. Considering that licarin A exhibited a considerable molecular complexity given by the asymmetry of the dihydrobenzofurane heterocycle, a molecular simplification approach was also applied in its structure (Fig. 2).

**Figure 3:**
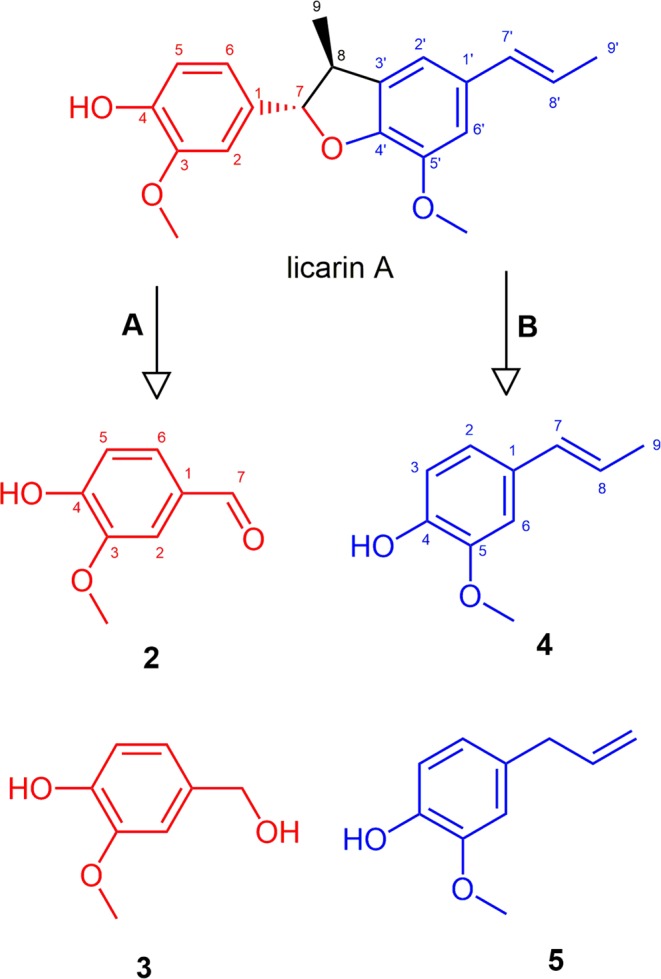
Molecular simplification approach to design the analogues **2–5** of licarin A (**1**). Note that licarin is comprised by a vanillin-like subunit (red – part A) and an isoeugenol-like subunit (blue – part B), which was explored in the search for the pharmacophore in licarin A.

Licarin A and its semi-synthetic analogues were designed to check the role of the phenolic hydroxyl in the biological activity of the natural product. However, licarin A present reduced solubility, that would impair the pharmacokinetic properties of this molecule *in vivo*. Considering that the semi-synthetic analogues **1a–1e** present modifications that lead to increased lipophilicity (as noted by the log P values – Table 1), it is expected that these compounds would present a solubility in water even worse than the natural prototype, and thus analogues with improved solubility profile should be considered. The simplification strategy has been widely used to make complex molecules with promising activity simpler, which allows keeping the pharmacophore unit in the analogue but with better drug-like properties (solubility and ADME profile), as well as providing compounds with simpler structure and cheaper obtaining process^22,23^. Moreover, these simplified analogues would keep or improve the antitrypanosomal activity of licarin A, but with better drug-likeness and water solubility. Accordingly, simplified analogues of vanillin (**2**), vanillyl alcohol (**3**), isoeugenol (**4**), and eugenol (**5**) were prepared by using the same modifications performed to licarin A, generating the corresponding analogues **a – d** (Fig. 3) which were chemically characterized by analysis of NMR (^1^H and ^13^C) and HRESIMS data.

**Table 1:** Anti-*T. cruzi* activity, cytotoxicity in mammalian cells, selectivity index, log P estimation, and apparent permeability for the licarin A, semisynthetic derivatives, simplified analogues, and positive control benznidazole (Bnz).

	IC_50_ (µM)		CC_50_ (µM)	SI		Clog P	Papp (10^−6^)	
	trypo	Ama	NCTC	trypo	ama		GIT	BBB
**1**	54.3 ± 8.9	>100	>200	>3.7	—	4.51	0.85	2.37
**1a**	28.0 ± 10.2	>100	>200	>7.1	—	4.65	0.42	4.26
**1b**	17.9 ± 2.9	>100	67.2 ± 24.6	3.7	—	4.42	1.02	0.34
**1c**	>100	>100	>200	—	—	5.39	0.43	0.24
**1d**	5.0 ± 0.8	>100	45.5 ± 16.7	9.0	—	5.61	0.38	0.28
**1e**	10.5 ± 5.7	>100	>200	>19.0	—	5.17	0.55	0.23
**2**	>100	5.5 ± 0.6	>200	—	>36.4	1.22	1.41	6.15
**2a**	>100	>100	>200	—	—	1.37	20.70	7.00
**2b**	>100	5.6 ± 0.3	>200	—	>35.6	1.14	17.40	4.70
**2c**	>100	>100	>200	—	—	2.10	1.60	5.35
**2d**	>100	>100	>200	—	—	2.32	1.57	1.25
**3**	>100	>100	>200	—	—	0.74	0.18	0.64
**3a**	>100	>100	>200	—	—	0.89	2.22	2.18
**3b**	5.1 ± 0.7	>100	45.8 ± 14.9	9.0	—	0.66	5.07	3.67
**3c**	>100	>100	>200	—	—	1.62	1.90	4.74
**3d**	>100	>100	>200	—	—	1.84	3.47	3.47
**4**	50.7 ± 17.4	>100	>200	>4.0	—	2.64	6.17	1.60
**4a**	8.8 ± 2.0	>100	>200	>25.0	—	2.78	15.90	1.29
**4b**	54.6 ± 10.2	>100	>200	>3.7	—	2.55	15.00	1.32
**4c**	>100	>100	>200	—	—	3.51	0.13	0.20
**4d**	21.2 ± 3.7	10.4 ± 0.9	>200	>9.5	>19.1	3.73	1.20	0.74
**5**	>100	>100	>200	—	—	2.61	14.40	3.98
**5a**	>100	>100	>200	—	—	2.76	2.35	1.56
**5b**	>100	>100	>200	—	—	2.52	2.81	2.28
**5c**	>100	>100	>200	—	—	3.49	1.21	0.32
**5d**	>100	>100	>200	—	—	3.71	0.65	0.32
**Bnz**	5.5 ± 0.9	18.7 ± 2.6	>200	>36.4	>10.7	—	—	—

### Antitrypanosomal activity

The antitrypanosomal potentials of the semi-synthetic analogues **1a, 1b, 1d** and **1e** were superior to the natural licarin A (**1**), as showed in Table 1. However, these compounds showed appreciable activity only against the trypomastigote form and were not active against the intracellular amastigote. The DNDi states that a good antichagasic agent should present activity against both forms of the parasite^5,9^. Efficacy against trypomastigotes also contributes on the clinical outcome, since this avoids the increases in parasitaemia that can contribute to the spreading of the parasites into healthy cells^9^. On the other hand, several compounds showed no important mammalian cytotoxicity to the maximal tested concentration of 200 μM, and even though some analogues showed some toxicity to the cells, the selectivity index (SI) were >19 for the intracellular amastigotes stage of the parasite, making these compounds interesting hits for further studies.

The most active semi-synthetic compound in the series was **1d**, which showed an IC_50_ of 5.0 ± 0.8 µM against the trypomastigotes, followed by compound **1e** (IC_50_ of 10.5 ± 5.7 µM). This suggests that the presence of an additional substitution in the aromatic ring of licarin A contributed to the antitrypanosomal activity. However, the presence of the phenolic hydroxyl led to increased cytotoxicity to mammalian cells, as can be observed in the results for compound **1d** (CC_50_ of 45.5 ± 16.7 μM). Conversely, moderate cytotoxicity was detected to acetyl derivative **1b** (CC_50_ 67.2 ± 24.6 μM). This can be attributed to the hydrolysis by cellular esterases, which can hydrolyse **1b** to the phenol **1** after its penetration into the cells, thus exerting cytotoxic effect.

Compounds with more stable substituents in the hydroxyl group (such as **1a**, **1c** and **1d**) showed no important cytotoxic effect against NCTC cells. This corroborates to our hypothesis on the toxicophoric role of the phenolic hydroxyl, and thus this functional group should be avoided to increase the selectivity of such compounds. However, some of the cytotoxicity can also be attributed to the excessive lipophilicity of such compounds. It is known that lipophilicity affects not only the water solubility of the drugs, but also the ADME properties and accordingly the toxicity^24,25^. Highly lipophilic compounds (log P > 5) tend to bind to hydrophobic sites in the cells, increasing the promiscuity and the cytotoxicity. Considering this aspect, analogues with reduced lipophilicity but maintaining the probable pharmacophore of licarin A were designed. Our pharmacophore hypothesis regards on the presence of the vanillin-like (Fig. 3, red) or the isoeugenol-like (Fig. 3, blue) motifs. Thus, simplified analogues from vanillin (**2**), vanillyl alcohol (**3**), isoeugenol (**4**), and eugenol (**5**) may present the pharmacophore motifs with less lipophilicity and improved water solubility indeed. The results presented in Table 1 show that analogues **2**, **2b**, **3b**, **4**, **4a**, **4b** and **4d** kept the pharmacophore to exert antiparasitic activity against the trypomastigote form, and some of them (**2**, **2b** and **4d**) also presented activity against the amastigote form of the parasite. Moreover, almost all compounds showed no relevant toxicity for the mammalian cells (except for compound **3b**), reinforcing the hypothesis of the contribution of excessive lipophilicity on the cytotoxic effect.

**Figure 4:**
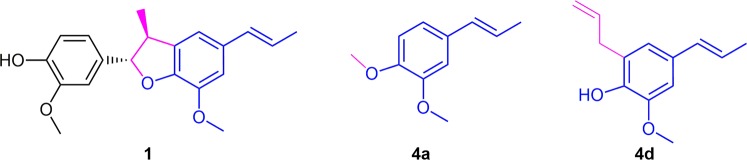
Possible pharmacophore fragment on licarin A (**1**) structure (blue) suggested by the activity observed to isoeugenol derivatives **4a** and **4d**, and the auxophore moieties (magenta).

Isoeugenol derivatives (**4a–4d**) showed the best activity profile against trypomastigote forms, with IC_50_ values ranging from 54.6 ± 10.2 to 8.8 ± 2.0 µM and thus yielding good selectivity towards the parasites. Among them, compound **4d** can be highlighted, since it also presented activity against the amastigotes (IC_50_ of 10.4 ± 0.9 µM) and a high SI value (>9.5). This result suggests that a possible pharmacophore fragment on licarin A structure is the isoeugenol moiety, as showed in Fig. 4. Furthermore, this data is corroborated by the poor activity showed by the eugenol derivatives^5^, on which the isomerization of the unsaturation of the terminal carbon was detrimental to the activity and thus this double bond seems to be part of the pharmacophore. Additionally, the substitution of the hydroxyl on isoeugenol (**4**) by a methyl group (**4a**) and the *ortho*-substitution in the aromatic ring (**4d**) seems to increase the antiparasitic activity. As shown in Table 1, compound **4c** was the unique isoeugenol derivative that did not display activity, suggesting that the presence of the allyloxy group is detrimental to the activity. The same is also observed for the compound **1c**.

Vanillin (**2**) and its acetyl derivative **2b** showed interesting activity against the amastigotes of *T. cruzi*. Despite the high activity presented by such compounds, this effect may be due the presence of the aldehyde, which is a reactive group and may exert antiparasitic effect through covalent interaction with parasitic proteins. Moreover, compound **2b** reinforces the hypothesis of a hydrolysis-dependent effect in the intracellular environment, since compounds **2** and **2b** showed the same activity. The explanation to the activity of such compounds only at amastigotes can be related to the higher activity of aldehyde reductases in the extracellular form of the parasite. Sanchez-Moreno and co-workers showed that epimastigotes (but not amastigotes) of *T. cruzi*, release ethanol to the environment^26^. Cazzulo *et al*. reported differences in the metabolism of the different forms of *T. cruzi*, suggesting that the extracellular forms present higher aldehyde reductase activity than the amastigotes^27^, showing that benzaldehydes are reduced faster than the benzyl alcohols are oxidized by the parasitic enzymes^28^. Considering that the corresponding alcohols were inactive in both forms of the parasite, the higher aldehyde reduction rate in the trypomastigotes may explain the reason for the activity of the aldehydes only against the amastigotes^29^.

### Mechanism of action studies

**Figure 5:**
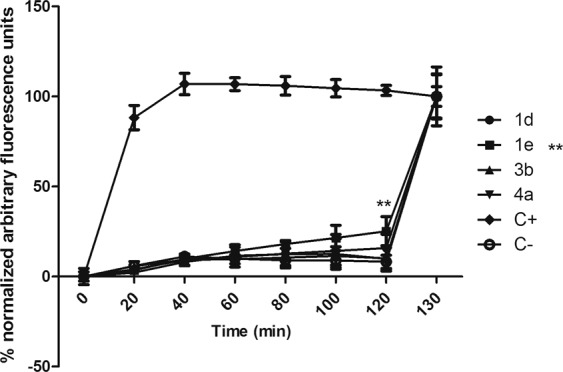
Plasma membrane permeability analysis on *T. cruzi* trypomastigotes with the probe Sytox Green treated with compounds **1d**, **1e**, **3b** and **4a** at the respective IC_50_ values. As positive (C+) and negative (C−) controls were used trypomastigotes treated with TX-100 at 0.5% (maximum permeabilization) and untreated *T. cruzi* parasites (minimum permeabilization), respectively. One representative experiment of two assays is shown. **p < 0.005.

**Figure 6:**
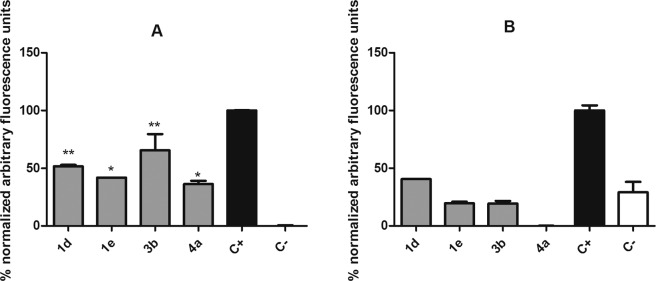
Evaluation of reactive oxygen species (ROS) generation in *T. cruzi* trypomastigotes treated with compounds **1d**, **1e**, **3b** and **4a** for 1 h (**A**) and 2 h (**B**). The H_2_DCFDA fluorescent probe was analyzed spectrofluorimetrically (excitation 485 nm and emission 520 nm). Untreated trypomastigotes and treated with azide (10 mM) were used to achieve minimal and maximal ROS production, negative (C−) and positive control (C+), respectively. One representative experiment of two assays is shown. **p  <  0.001.

**Figure 7:**
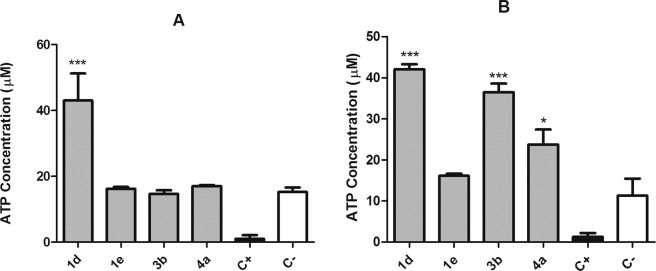
Evaluation of ATP production in *T. cruzi* trypomastigotes treated by 1 h (**A**) and 2 h (**B**) with the compounds **1d**, **1e**, **3b** and **4a** (IC_50_ values). Untreated trypomastigotes, negative control (C−) and treated with CCCP (100 µM), positive control (C+) were used as controls of minimal and maximal depolarization. One representative experiment of two assays is shown. ***p < 0.0001.

**Figure 8:**
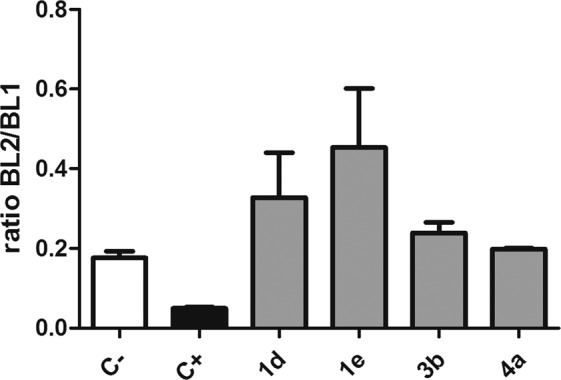
Mitochondrial membrane potential analysis in *T. cruzi* trypomastigotes treated with compounds **1d**, **1e**, **3b** and **4a** for two hours labeled with JC-1 probe (0.2 μM). The fluorescence was measured in a flow cytometer (ATTUNE). Minimum (untreated – negative control, C−) and maximum (treated with CCCP- 100 µg/mL - positive control, C+) alterations in the mitochondrial membrane potential were obtained. Fluorescence was quantified by calculating the ratio between the channels BL2/BL1. One representative experiment of two assays is shown.

Compounds **1d, 1e, 3b**, and **4a** exhibited higher activity against *T. cruzi* trypomastigotes and were selected for studies of mechanism of action, to understand the possible alterations caused by these compounds in the plasma membrane permeability and the mitochondria of *T. cruzi* trypomastigotes. The fluorescent probe Sytox Green enters damaged cells and binds to nucleic acid, increasing 500-fold the fluorescence levels. Alterations in the plasma membrane can lead to a total parasite breakdown affecting morphology, fluidity, ion transport, and consequent cell death^30^. In our study, no changes in the fluorescence levels were observed for all the studied compounds, with exception of **1e** after 120 min of incubation (Fig. 5) when compared to untreated parasites. The production of reactive oxygen species (ROS) was determined with the cell permeant fluorescence probe H_2_DCFDA. After 1 h of treatment with all studied compounds, it was possible to observe an increased ROS generation by the trypomastigotes, followed by a drop of the levels after 2 h (Fig. 6A,B), showing the cellular apparatus controlling the oxidative stress caused by these highly toxic radicals. The redox imbalance occurs when the endogenous antioxidants fail to remove the excessive ROS produced, which leads to oxidative stress^31^. The bioenergetic system of the parasite was highly compromised, as a result of the increased ATP production after 2 h of incubation with all compounds. This energy expenditure to control the homeostasis was clearly observed in the parasites incubated with all compounds, with exception of compound **1e**, which induced no significant alterations in ATP levels (Fig. 7). The stability of ATP levels and mitochondrial membrane potential is a requisite for a normal cell functioning^32^. Mitochondria is a single organelle in trypanosomatids and is directly involved in redox status of the parasite^33^ and plays a central role in energy metabolism, being the site of the oxidative phosphorylation that drives the ATP synthesis and represent the main sources of ROS. Additionally, it participates in the nutrient oxidation, calcium homeostasis and apoptosis^34^. With the exception of compound **4a**, the compounds induced a mitochondrial hyperpolarization, but with no statistical significance (Fig. 8), suggesting that they probably lead to alterations in the respiratory chain.

### Permeability of licarin A and analogues in PAMPA models

To estimate the intestinal absorption and permeability of the semi-synthetic and simplified analogues of licarin A through the BBB, two PAMPA models were employed, as summarized in Table 1. Accordingly, the semi-synthetic derivatives **1a–1e** present low intestinal permeability since their Papp values are lower than 1.0 × 10^−6^ cm/s, suggesting that the excessive lipophilicity may impair the oral bioavailability of these compounds. This result is in agreement with the low permeability observed in the BBB model for **1b – 1e**. The Lipinski’s rule-of-five^35^ defines that compounds with high molecular mass (>500 Da) and high log P (>5) may present low oral bioavailability, so the compounds fulfil the rule-of-five. However, they already present Clog P values close to 5, and further modifications would raise this value over 5. The rule-of-three^36^ is used as guide for designing compounds identified from screening tests because it considers that medicinal chemists will modify the compound, increasing the molecular weight and the lipophilicity. Accordingly, the threshold of rule-of-three is more rigid, limiting the log P until 3 and molecular mass until 300 and thus compound **1** do not fulfil this criterion. Considering this point, the simplified compounds **2**–**5** are smaller, less lipophilic compounds that fulfil the rule-of-three and, as can be noted in the results from Table 1, these compounds presented increased permeability through GIT and limited permeability through BBB. This data suggests that these compounds present better drug-likeness, improved oral bioavailability and less CNS-related off-target toxicity. Therefore, the most promising was the compound **4**, which presented adequate permeability on GIT model, and did not seem to cross the BBB, reinforcing its promising pharmacological profile. In addition, the PAMPA-GIT model also estimated that compound **4** has a protein plasma binding percentage lower than 90% (since Papp is <1 × 10^−5^ cm/s)^37^, which is desirable from the clinical point-of-view. In counterpart, lipophilic compounds such as **2a**, **2b**, **4a**, **4b** and **5** seem to have a plasma protein binding percentage higher than 90% (Table 1) which can impair the bloodstream availability of the free compound and their distribution into the tissues, where the amastigotes are present, compromising drug efficacy.

## Conclusions

In summary, although the semi-synthetic derivatives of licarin A showed activity against *T. cruzi*, their low drug-likeness limit their exploitation as prototypes for designing novel compounds with improved pharmacological profile. Therefore, the molecular simplification approach increased the lead-likeness of the set and generated fewer complex compounds with interesting antiparasitic activity that can be considered better prototypes for further modifications, aiming improved activity allied with promising ADME profile.

## Methods

### General experimental procedures

^1^H and ^13^C NMR spectra were recorded on a Bruker Advance 300, operating at 300 MHz for ^1^H and 75 MHz for ^13^C, using CDCl_3_ as solvent and TMS as internal standard. Chemical shifts (δ) are given in ppm and the coupling constants (*J*) are presented in Hz. HRESIMS spectra were measured on a Bruker Daltonics MicroTOF QII spectrometer. Starting materials were acquired from commercial suppliers with purity higher than 98% and used without further processing. Claisen rearrangements were performed on a Discovery microwave reactor (CEM Inc.) using a sealed reaction vial with a high-pressure accessory. Column chromatography (CC) procedures were performed using silica gel 60 while progress of the reactions was monitored through TLC in silica gel plates with fluorescence indicator and visualized at 254 nm.

### Plant material

Fresh leaves of *Nectandra oppositifolia* were collected at Artur Nogueira city, São Paulo State, Brazil (22°30′57,65″S and 47°10′50,11″W) in April/2016. The plant material was identified by Prof. MSc. Guilherme M. Antar. A voucher specimen was compared with that under code SPF225339, deposited in the Herbarium of Institute of Biosciences, University of São Paulo, SP, Brazil.

### Isolation of licarin A from *N. oppositifolia*

After being dried and powdered, the leaves of *N. oppositifolia* (332 g) were extracted using *n*-hexane (6 × 1 L) at room temperature. After evaporation of the solvent at reduced pressure, 32 g of crude *n*-hexane extract were obtained. Part of this material (20 g) was chromatographed over a silica gel column eluted with *n*-hexane containing increasing amounts of EtOAc to afford five groups (I–V). Part of the group IV (3280 mg) was chromatographed over a silica gel column eluted with mixtures of *n*-hexane:EtOAc (8:2, 7:3, 1:1, and 2:8) to afford 1280 mg of licarin A.

*Licarin A* (**1**). ^1^H NMR (CDCl_3_), δ 6.97 (d, *J* = 1.4 Hz, H-2), 6.92 (dd, *J* = 7.8 and 1.4 Hz, H-6), 6.91 (d, *J* = 7.8 Hz, H-5), 6.79 (br s, H-2′), 6.77 (br s, H-6′), 6.36 (dd, *J* = 15.7 and 1.6 Hz, H-7′), 6.11 (dq, *J* = 15.7 and 6.6 Hz, H-8′), 5.63 (s, OH), 5.11 (d, *J* = 9.4 Hz, H-7), 3.90 (s, OCH_3_), 3.89 (s, OCH_3_), 3.45 (dq, *J* = 9.4 and 6.8 Hz, H-8), 1.87 (dd, *J* = 6.6 and 1.6 Hz, H-9′), 1.38 (d, *J* = 6.8 Hz, H-9). ^13^C NMR (CDCl_3_), δ 146.7 (C-3), 146.6 (C-4′), 145.8 (C-4), 144.2 (C-5′), 132.2 (C-1′), 132.1 (C-1), 130.9 (C-7′), 123.5 (C-8′), 120.0 (C-6), 114.1 (C-5), 113.3 (C-2′), 113.2 (C-3′), 109.3 (C-6′), 108.9 (C-2), 93.8 (C-7), 56.1 (OCH_3_), 56.0 (OCH_3_), 45.6 (C-8), 18.4 (C-9′), 17.6 (C-9). HRESIMS *m/z* 327.1592 [M + H]^+^ (calcd for C_20_H_23_O_4_ 327.1596).

### Molecular simplification of licarin A and derivatives

The molecular simplification approach was employed to licarin A and semi-synthetic derivatives considering that licarin A is comprised by a vanillin-like moiety (represented in red – part A, in Fig. 3) and an isoeugenol-like moiety (represented in blue – part B, in Fig. 3). The parent licarin molecule was then broke apart to generate the vanillin analogues (**2** and **3**) and the isoeugenol analogues (**4** and **5**), with the same substitution pattern from the semi-synthetic derivatives (**a** – 4-methoxy; **b** – 4-acetoxy; **c** – 4-allyloxi; **d** – 5-allyl). It must be in mind that simplification strategy is very intuitive and based on a medicinal chemist’s hypothesis from the pharmacophore units of the parent molecule, that is usually supported by preliminary SAR data^22,23^, as those obtained with the derivatives of licarin A.

### General procedure for the preparation of compounds 1a–5a

Using individual flasks containing licarin A (**1**), vanillin (**2**), vanillyl alcohol (**3**), isoeugenol (**4**), or eugenol (**5**), two equivalents of K_2_CO_3_, two equivalents of methyl iodide and 10 mL of acetone were added. The reaction mixtures were stirred under reflux for 7 h, and thus the volatiles were evaporated under reduced pressure. The residue was taken up in CH_2_Cl_2_ and washed with H_2_O (2 × 25 mL). The organic layers were separated, dried over anhydrous Na_2_SO_4_ and the solvent was evaporated under reduced pressure. Crude products were purified through silica gel column chromatography using *n*-hexane:EtOAc (9:1) as eluent.

*(2*S*,3 *S*)-2-(3,4-dimethoxyphenyl)-7-methoxy-3-methyl-5-[(*E*)-prop-1-enyl]-2,3-dihydrobenzo-furan (****1a****):* 220 mg of compound **1** yielded 73% of **1a** as a white amorphous solid. ^1^H NMR (CDCl_3_) δ 7.01 (d, *J* = 1.7 Hz, H-5); 7.00 (dd, *J* = 6.0 and 1.8 Hz, H-6), 6.95 (d, *J* = 1.9 Hz, H-2), 6.86 (d, *J* = 8.2 Hz, H-6′), 6.80 (d, *J* = 6.3 Hz, H-2′), 6.36 (dd, *J* = 15.6 and 1.3 Hz, H-7′), 6.10 (dq, *J* = 15.6 and 6.5 Hz, H-8′), 5.11 (d, *J* = 9.4 Hz, H-7), 3.91 (s, OCH_3_), 3.90 (s, OCH_3_), 3.88 (s, OCH_3_), 3.45 (dd, *J* = 9.2 and 6.8 Hz, H-8), 1.86 (dd, *J* = 6.6 and 1.3 Hz, H-9′), 1.38 (d, *J* = 6.8 Hz, H-9). ^13^C NMR (CDCl_3_) δ 149.1 (C-4), 149.0 (C-5′), 146.6 (C-6′), 144.2 (C-3), 133.3 (C-4′), 132.6 (C-6), 132.2 (C-6′), 130.9 (C-7′), 123.5 (C-8′), 119.2 (C-1), 113.3 (C-2), 110.8 (C-3′), 109.5 (C-1′), 108.9 (C-5), 93.6 (C-7), 55.9 (OCH_3_), 55.8 (OCH_3_), 55.9 (OCH_3_), 45.5 (C-8), 18.4 (C-9′), 17.6 (C-9). HRESIMS *m/z* 341.1750 [M + H]^+^ (calcd. for C_21_H_25_O_4_ 341.1753).

*3,4-dimethoxybenzaldehyde (****2a****):* 250 mg of compound **2** yielded 70% of **2a** as white crystals. ^1^H NMR (CDCl_3_) δ 9.61 (s, H-7), 7.44 (dd, *J* = 8.0 and 1.8 Hz, H-6), 7.33 (s, *J* = 1.8 Hz, H-2), 7.18 (d, *J* = 8.0 Hz, H-5), 3.87 (s, OCH_3_), 3.83 (s, OCH_3_). ^13^C NMR (CDCl_3_) δ 191.0 (C-7), 155.8 (C-4), 150.7 (C-3), 130.4 (C-1), 127.1 (C-6), 115.4 (C-5), 109.9 (C-2), 56.1 (OCH_3_), 56.0 (OCH_3_). HRESIMS *m/z* 167.0713 [M + H]^+^ (calcd. for C_9_H_11_O_3_ 167.0708).

*(3,4-dimethoxyphenyl)methanol (****3a****):* 270 mg of compound **3** yielded 66% of **3a** as yellowish liquid. ^1^H NMR (CDCl_3_) δ 6.86 (br s, H-6 and H-5), 6.83 (s, H-2), 3.82 (s, OCH_3_). ^13^C NMR (CDCl_3_) δ 191.0 (C-7), 155.8 (C-4), 150.7 (C-3), 130.4 (C-1), 127.1 (C-6), 115.4 (C-5), 109.9 (C-2), 56.1 (OCH_3_), 55.9 (OCH_3_). HRESIMS *m/z* 169.0870 [M + H]^+^ (calcd. for C_9_H_13_O_3_ 169.0865).

*1,2-dimethoxy-4-[(*E*)-prop-1-enyl]benzene (****4a****):* 280 mg of compound **4** yielded 59% of **4a** as yellowish liquid. ^1^H NMR (CDCl_3_) δ 6.65 (dd, *J* = 8.0 Hz, H-6), 6.48 (d, *J* = 8.0 Hz, H-2), 6.44 (dd, *J* = 15.1 and 0.8 Hz, H-7), 6.30 (d, *J* = 8.0 and 1.9 Hz, H-6), 6.00–5.88 (m, H-8), 3.85 (s, OCH_3_), 3.83 (s, OCH_3_), 1.97 (dd, *J* = 6.3 and 0.8 Hz, H-9). ^13^C NMR (CDCl_3_) δ 153.8 (C-2), 150.7 (C-1), 150.4 (C-6), 137.1 (C-8), 135.4 (C-4), 115.9 (C-9), 111.7 (C-5), 107.4 (C-3), 56.1 (OCH3), 56.0 (OCH_3_), 39.9 (C-7). HRESIMS *m/z* 179.1069 [M + H]^+^ (calcd. for C_11_H_15_O_2_ 179.1072).

*4-allyl-1,2-dimethoxy-benzene (****5a****):* 290 mg of compound **5** yielded 65% of **5a** as citrine liquid. ^1^H NMR (CDCl_3_) δ 6.68 (dd, *J* = 8.0 and 1.9 Hz, H-6), 6.45 (d, *J* = 1.9 Hz, H-2), 6.30 (d, *J* = 1.9 Hz, H-5), 6.04–5.83 (m, H-8), 5.10–4.99 (m, H-9), 3.85 (s, OCH_3_), 3.83 (s, OCH_3_), 3.24–3.22 (m, H-7). ^13^C NMR (CDCl_3_) δ 153.8 (C-2), 150.7 (C-1), 150.4 (C-6), 137.1 (C-8), 135.4 (C-4), 115.9 (C-9), 111.7 (C-5), 107.4 (C-3), 56.1 (OCH_3_), 56.0 (OCH_3_), 39.9 (C-7). HRESIMS *m/z* 179.1070 [M + H]^+^ (calcd. for C_11_H_15_O_2_ 179.1072).

### General procedure for the preparation of compounds 1b – 5b

Using individual flasks containing compounds **1–5**, one equivalent of triethylamine, two equivalents of acetyl chloride and 5 mL of CH_2_Cl_2_ were added. The reaction mixtures were stirred under an ice bath for 4 h, when NaHCO_3_ solution (10%) was added for neutralization of acids. The organic layers were separated, washed with H_2_O (2 × 25 mL), dried over anhydrous Na_2_SO_4_ and evaporated under reduced pressure. Crude products were purified through silica gel column chromatography using *n*-hexane:EtOAc (9:1) as eluent.

*[2-methoxy-4-[(2*S*,3 *S*)-7-methoxy-3-methyl-5-[(*E*)-prop-1-enyl]-2,3-dihydrobenzofuran-2-yl]phenyl] acetate (****1b****):* 350 mg of compound **1** yielded 27% of **1b** as white amorphous solid. ^1^H NMR (CDCl_3_) δ 7.00 (br s, H-6′), 6.98 (br s, H-2′), 6.88 (br s, H-2), 6.80 (br s, H-6), 6.78 (br s, H-5), 6.36 (dd, *J* = 15.7 and 1.5 Hz, H-7′), 6.10 (dq, *J* = 15.7 and 6.6 Hz, H-8′), 5.11 (d, *J* = 9.2 Hz, H-7), 3.46 (dd, *J* = 9.2 and 6.8 Hz, H-8), 1.87 (dd, *J* = 6.6 and 1.5 Hz, H-9′), 1.41 (d, *J* = 6.8 Hz, H-9), 3.90 (s, OCH_3_), 3.88 (s, OCH_3_), 2.31 (s, CH_3_). ^13^C NMR (CDCl_3_) δ 169.0 (C = O), 151.2 (C-5′), 146.5 (C-4), 144.1 (C-6′), 139.6 (C-3), 139.3 (C-4′), 133.0 (C-6), 132.4 (C-6′), 130.9 (C-7′), 123.6 (C-8′), 122.7 (C-1), 118.7 (C-2), 113.4 (C-3′), 110.3 (C-1′), 109.3 (C-5), 93.1 (C-7), 55.9 (OCH_3_), 45.8 (C-8), 17.9 (C-9), 20.7 (CH_3_), 18.4 (C-9′), HRESIMS *m/z* 369.1707 [M + H]^+^ (calcd. for C_22_H_25_O_5_ 369.1702).

*(4-formyl-2-methoxy-phenyl) acetate (****2b****):* 380 mg of compound **2** yielded 38% of **2b** as white crystals. ^1^H NMR (CDCl_3_) δ 9.90 (s, H-7), 7.46 (d, *J* = 1.8 Hz, H-2), 7.44 (dd, *J* = 8.0 and 1.8 Hz, H-6), 7.20 (d, *J* = 8.0 Hz, H-5), 3.82 (s, OCH_3_), 2.29 (s, CH_3_). ^13^C NMR (CDCl_3_) δ 191.0 (C-7), 169.0 (C = O), 151.5 (C-3), 140.7 (C-4), 125.9 (C-1), 122.8 (C-5), 121.1 (C-6), 113.6 (C-2), 55.8 (OCH_3_), 20.3 (CH_3_). HRESIMS *m/z* 195.0663 [M + H]^+^ (calcd. for C_10_H_11_O_5_ 195.0657).

*[4-(hydroxymethyl)-2-methoxy-phenyl] acetate (****3b****):* 350 mg of compound **3** yielded 27% of **3b** as yellow liquid. ^1^H NMR (CDCl_3_) δ 7.02 (br s, H-5), 7.00 (br s, H-6), 6.83 (br s, H-2), 4.73 (s, H-7) 3.81 (s, OCH_3_) 2.28 (s, CH_3_). ^13^C NMR (CDCl_3_) δ 191.0 (C-7), 169.1 (C = O), 152.2 (C-3), 147.7 (C-4), 134.7 (C-1), 125.1 (C-5), 124.4 (C-6), 112.9 (C-2), 55.6 (OCH_3_), 20.1 (CH_3_). HRESIMS *m/z* 197.0810 [M + H]^+^ (calcd. for C_10_H_13_O_4_ 197.0814).

*[2-methoxy-4-[(*E*)-prop-1-enyl]phenyl] acetate (****4b****):* 380 mg of compound **4** yielded 67% of **4b** as yellow liquid. ^1^H NMR (CDCl_3_) δ 6.97 (d, *J* = 8.0 Hz, H-5), 6.92 (d, *J* = 8.0 Hz, H-6), 6.91 (br s, H-2), 6.48 (dd, *J* = 15.1 Hz, H-7), 6.25–6.18 (m, H-8), 3.85 (s, OCH_3_), 2.28 (s, CH_3_), 1.85 (3 H, dd, *J* = 6.0 and 0.8 Hz, H-9). ^13^C NMR (CDCl_3_) δ 169.0 (C = O), 151.5 (C-3), 141.1 (C-4), 136.5 (C-1), 130.5 (C-7), 124.4 (C-5), 124.0 (C-8), 121.9 (C-6), 55.8 (OCH_3_), 20.1 (CH_3_), 18.8 (C-9). HRESIMS *m/z* 207.1018 [M + H]^+^ (calcd. for C_12_H_15_O_3_ 207.1021).

*(4-allyl-2-methoxy-phenyl) acetate (****5b****):* 380 mg compound **5** yielded 69% of **5b** as yellow liquid. ^1^H NMR (CDCl_3_) δ 7.06 (dd, *J* = 8.0 and 2.0 Hz, H-6), 7.01 (d, *J* = 8.0 Hz, H-5), 6.98 (d, *J* = 2.0 Hz, H-2), 6.22–6.15 (m, H-8), 5.10–5.02 (m, H-9), 3.80 (s, OCH_3_), 3.33 (d, *J* = 6.2 Hz, H-7), 2.28 (3 H, s, CH_3_). ^13^C NMR (CDCl_3_) δ 169.4 (C = O), 151.8 (C-3), 138.7 (C-4), 137.4 (C-1), 136.1 (C-8), 122.4 (C-5), 121.9 (C-6), 115.7 (C-9), 55.8 (OCH_3_), 39.8 (C-7), 20.5 (CH_3_). HRESIMS *m/z* 207.1020 [M + H]^+^ (calcd. for C_12_H_15_O_3_ 207.1021).

### General procedure for the preparation of compounds 1c – 5c

Using individual flasks containing compounds **1–5**, two equivalents of K_2_CO_3_, two equivalents of allyl bromide and 15 mL of acetone were added. The reaction mixtures were stirred under reflux for 19 h, and thus the volatiles were evaporated under reduced pressure. The residues were dissolved in CH_2_Cl_2_ and washed with H_2_O (2 × 25 mL). The organic layers were separated, dried over anhydrous Na_2_SO_4_ and evaporated under reduced pressure. Crude products were purified through silica gel column chromatography using *n*-hexane:EtOAc (9:1) as eluent.

*(2*S*,3 *S*)-2-(4-allyloxy-3-methoxy-phenyl)-7-methoxy-3-methyl-5-[(*E*)-prop-1-enyl]-2,3-dihydrobenzofuran (****1c****):* 600 mg of compound **1** yielded 73% of **1c** as white amorphous solid. ^1^H NMR (CDCl_3_) δ 6.99 (d, *J* = 8.2 Hz, H-5), 6.93 (d, *J* = 1.9 Hz, H-2), 6.92 (dd, *J* = 8.2 and 1.9 Hz, H-6), 6.84 (d, *J* = 1.9 Hz, H-2′), 6.78 (d, *J* = 1.9 Hz, H-6′), 6.36 (dd, *J* = 15.7 and 1.5 Hz, H-7′), 6.09 (qdd, *J* = 10.6, 7.2 and 4.8 Hz, H-β), 6.04–6.16 (m, H-8′), 5.39 (dd, *J* = 17.3 and 1.5 Hz; H-γ), 5.11 (d, *J* = 9.4 Hz, H-7), 4.61 (dt, *J* = 5.4 and 1.4 Hz, H-α), 3.86 (s, OCH_3_), 3.89 (s, OCH_3_), 3.46 (dq, *J* = 13.6 and 6.8 Hz; H-8), 1.86 (dd, *J* = 6.6 and 1.3 Hz, H-9′), 1.38 (d, *J* = 6.8 Hz, H-9). ^13^C NMR (CDCl_3_) δ 149.6 (C-5′), 148.1 (C-4′), 146.6 (C-6′), 144.1 (C-3), 133.3 (C-4), 133.2 (C-2), 133.0 (C-6), 132.3 (C-2′), 130.9 (C-7′), 123.5 (C-8′), 119.1 (C-1), 118.0 (C-γ), 113.3 (C-β), 113.1 (C-3′), 109.9 (C-1′), 109.2 (C-5), 93.6 (C-7), 69.9 (C-α), 55.9 (OCH_3_), 45.6 (C-8), 18.4 (C-9′), 17.7 (C-9), HRESIMS *m/z* 367.1920 [M + H]^+^ (calcd. for C_23_H_26_O_4_ 367.1909).

*4-allyloxy-3-methoxy-benzaldehyde (****2c****):* 700 mg of compound **2** yielded 77% of **2c** as yellowish liquid. ^1^H NMR (CDCl_3_) δ 9.88 (s, H-7), 7.33 (br s, H-2), 7.32 (br s, H-6), 7.02 (br s, H-5), 5.91–5.83 (m, H-β), 5.20–5.18 (m, H-γ), 4.61 (d, J = 6.2 Hz, H-α), 3.83 (s, OCH_3_). ^13^C NMR (CDCl_3_) δ 191.0 (C-7), 153.7 (C-4), 149.0 (C-3), 132.7 (C-β), 130.1 (C-1), 124.6 (C-6), 118.2 (C-γ), 108.9 (C-5), 70.0 (C-α), 55.3 (OCH_3_). HRESIMS *m/z* 193.0860 [M + H]^+^ (calcd. for C_11_H_13_O_3_ 193.0865).

*(4-allyloxy-3-methoxy-phenyl)methanol (****3c****):* 700 mg of compound **3** yielded 81% of **3c** as white solid. ^1^H NMR (CDCl_3_) δ 6.84 (br s, H-2), 6.75 (br s, H-6), 6.75 (br s, H-5), 6.08–5.96 (ddt, *J* = 16.5, 10.4 and 5.2 Hz, H-β), 5.35 (d, *J* = 16.0 Hz, H-γ), 4.51 (d, *J* = 5.3 H-α), 3.75 (s, OCH_3_). ^13^C NMR (CDCl_3_) δ 151.1 (C-4), 150.0 (C-3), 133.5 (C-β), 121.4 (C-1), 118.2 (C-γ), 117.6 (C-6), 112.6 (C-2 and C-5), 70.5 (C-α), 65.1 (C-7), 56.1 (OCH_3_). HRESIMS *m/z* 195.1019 [M + H]^+^ (calcd for C_11_H_15_O_3_ 195.1021).

*1-allyloxy-2-methoxy-4-[(*E*)-prop-1-enyl]benzene (****4c****):* 800 mg of compound **4** yielded 78% of **4c** as yellow liquid. ^1^H NMR (CDCl_3_) δ 6.87 (br s, H-6), 6.79 (br s, H-2), 6.48 (br s, H-5), 6.45 (dd, *J* = 15.0 and 1.0 Hz, H-7), 6.22 (m, H-8), 5.90–5.85 (m, H-β), 5.17 (m, H-γ), 4.60 (d, *J* = 6.2 Hz, H-α), 3.80 (s, OCH_3_), 1.77 (dd, *J* = 6.4 and 0.6 Hz, H-9). ^13^C NMR (CDCl_3_) δ 149.8 (C-3), 147.1 (C-4), 133.5 (C-β), 132.4 (C-1), 130.3 (C-7), 124.4 (C-8), 118.2 (C-γ), 119.8 (C-6), 112.1 (C-2 and C-5), 70.0 (C-α), 56.1 (OCH_3_), 18.7 (C-9). HRESIMS *m/z* 205.1224 [M + H]^+^ (calcd. for C_13_H_17_O_2_ 205.1229).

*4-allyl-1-allyloxy-2-methoxy-benzene (****5c****):* 800 mg of compound **5** yielded 88% of **5c** as yellow liquid. ^1^H NMR (CDCl_3_) δ 6.94 (d, *J* = 1.8 Hz, H-2), 6.89 (br s, H-5), 6.80 (br s, H-6), 6.23–6.19 (m, H-8), 5.90–5.82 (m, H-β), 5.21–5.12 (m, H-9), 4.60 (d, *J* = 6.1 Hz, H-α), 3.80 (s, OCH_3_), 3.33 (d, *J* = 6.2 Hz, H-7). ^13^C NMR (CDCl_3_) δ 149.8 (C-3), 149.5 (C-4), 136.5 (C-8), 133.1 (C-1 and C-β), 122.4 (C-6), 118.2 (C-γ), 115.9 (C-9), 114.1 (C-2), 112.3 (C-5), 70.5 (C-α), 56.1 (OCH_3_), 39.9 (C-7). HRESIMS *m/z* 205.1226 [M + H]^+^ (calcd. for C_13_H_17_O_2_ 205.1229).

### General procedure for the preparation of compounds 1d – 5d

Individual flasks containing compounds **1c–5c** dissolved in 3 mL of N,N-dimethylformamide were submitted to microwave irradiation (200 °C, 300 W, 300 psi) for 2 h. After evaporation of the solvent under reduced pressure, the crude products were dissolved in CH_2_Cl_2_ and washed with H_2_O (2 × 25 mL). The organic layers were dried over anhydrous Na_2_SO_4_ and evaporated under reduced pressure. Crude products were purified through silica gel column chromatography using *n*-hexane:EtOAc (9:1) as eluent.

*2-allyl-6-methoxy-4-[(2*S*,3 *S*)-7-methoxy-3-methyl-5-[(*E*)-prop-1-enyl]-2,3-dihydrobenzofuran-2-yl]phenol (****1d****):* 320 mg of compound **1c** yielded 53% of **1d** as white amorphous solid. ^1^H NMR (CDCl_3_) δ 6.90 (d, *J* = 1.6 Hz, H-6), 6.84 (d, *J* = 1.6 Hz, H-2), 6.83 (dd, *J* = 1.7 Hz, H-2′), 6.81 (d, *J* = 1.7 Hz, H-6′), 6.40 (dd, *J* = 15.7 and 1.3 Hz, H-7′), 6.36 (dd, *J* = 15.7 and 1.4 Hz, H-γ), 6.13–6.09 (m, H-β), 6.08–5.98 (m, H-8′), 5.73 (s, OH), 5.12 (br s, H-7), 3.87 (s, OCH_3_), 3.83 (s, OCH_3_), 3.54–3.34 (m, H-8), 3.45 (dd, *J* = 6.4 and 1.1 Hz, H-α), 1.86 (dd, *J* = 6.6 and 1.3 Hz, H-9′), 1.36 (d, *J* = 6.8 Hz, H-9). ^13^C NMR (CDCl_3_) δ 146.7 (C-3), 146.6 (C-4′), 144.2 (C-4), 143.5 (C-5), 136.5 (C-γ), 133.4 (C-3′), 132.2 (C-1), 131.2 (C-6′), 131.0 (C-7′), 125.5 (C-2′), 123.4 (C-8′), 121.0 (C-6), 115.6 (C-5′), 113.3 (C-1′), 109.3 (C-2), 107.0 (C-β), 94.0 (C-7), 56.1 (OCH_3_), 55.9 (OCH_3_), 45.5 (C-8), 33.9 (C−α), 18.4 (C-9′), 17.5 (C-9). HRESIMS *m/z* 367.1907 [M + H]^+^ (calcd. for C_23_H_27_O_4_ 367.1909).

*3-allyl-4-hydroxy-5-methoxy-benzaldehyde (****2d****):* 220 mg of compound **2c** yielded 49% of **2d** as white amorphous solid. ^1^H NMR (CDCl_3_) δ 9.80 (s, H-7), 7.31 (br s, H-6), 7.30 (br s, H-2), 6.07–5.93 (m, H-β). 5.14 (m, H-γ), 3.94 (s, OCH_3_), 3.46 (d, *J* = 5.0 Hz, H-α). ^13^C NMR (CDCl_3_) δ 197.1 (C-7), 151.1 (C-1), 148.1 (C-4), 147.0 (C-3), 136.5 (C-β), 130.9 (C-5), 126.5 (C-2), 115.9 (C-γ), 111.1 (C-6), 33.9 (C-α), 56.1 (OCH_3_). HRESIMS *m/z* 193.0861 [M + H]^+^ (calcd. for C_11_H_13_O_3_ 193.0865).

*2-allyl-4-(hydroxymethyl)-6-methoxy-phenol (****3d****):* 220 mg of compound **3c** yielded 18% of **3d** as white amorphous solid. ^1^H NMR (CDCl_3_) δ 7.03 (dt, *J* = 2.1 and 1.0 Hz, H-6), 6.76 (d, *J* = 2.1 Hz, H-2), 5.86 (tt, *J* = 13.4 and 6.2 Hz, H-β), 5.05 (dt, *J* = 13.4 and 1.0 Hz, H-γ), 4.46 (s, H-7), 3.86 (s, OCH_3_), 3.38 (dt, *J* = 6.2 and 1.0 Hz, H-α). ^13^C NMR (CDCl_3_) δ 146.9 (C-5), 144.6 (C-1), 136.5 (C-β), 132.7 (C-4), 130.1 (C-6), 124.6 (C-3), 123.3 (C-2), 113.8 (C-γ), 65.5 (C-7), 56.4 (OCH_3_), 33.4 (C-α). HRESIMS *m/z* 195.1023 [M + H]^+^ (calcd. for C_11_H_15_O_3_ 195.1021).

*2-allyl-6-methoxy-4-[(*E*)-prop-1-enyl]phenol (****4d****):* 300 mg of compound **4c** yielded 43% of **4d** as yellow liquid. ^1^H NMR (CDCl_3_) δ 6.90 (s, H-2), 6.71 (s, H-6), 6.54 (dd, *J* = 15.1 and 0.8 Hz, H-7), 6.24–6.21 (m, H-8), 6.20–6.18 (m, H-β), 5.08 (m, H-γ), 4.60 (d, *J* = 6.2 Hz, H-α), 3.83 (s, OCH_3_), 3.36 (d, *J* = 6.2 Hz, H-α), 1.90 (dd, *J* = 6.2 and 0.8 Hz, H-9). ^13^C NMR (CDCl_3_) δ 146.9 (C-5), 144.6 (C-4), 136.5 (C-β), 132.7 (C-1), 130.5 (C-7), 130.1 (C-3), 124.6 (C-8), 123.3 (C-2 and C-6), 113.8 (C-γ), 56.1 (OCH_3_), 33.2 (C-α), 18.9 (C-9). HRESIMS *m/z* 205.1223 [M + H]^+^ (calcd. for C_13_H_17_O_2_ 205.1229).

*2,4-diallyl-6-methoxy-phenol (****5d****):* 300 mg of compound **5c** yielded 39% of **5d** as yellow liquid. ^1^H NMR (CDCl_3_) δ 7.00 (br s, H-6), 6.92 (br s, H-2), 6.19 (m, H-β), 6.18 (m, H-8), 5.18 (m, H-γ), 5.04 (m, H-9), 3.84 (s, OCH_3_), 3.35 (m, H-7) 3.30 (d, *J* = 6.1 Hz, H-α). ^13^C NMR (CDCl_3_) δ 149.8 (C-5), 149.5 (C-4), 136.5 (C-8), 133.1 (C-β), 122.4 (C-6), 118.2 (C-γ), 115.9 (C-9), 114.1 (C-2), 112.3 (C-3), 56.1 (OCH_3_), 40.0 (C-α), 38.9 (C-7). HRESIMS *m/z* 205.1225 [M + H]^+^ (calcd. for C_13_H_17_O_2_ 205.1229).

### Synthesis of compound 1e

To 20 mL of a solution of **1d** (0.5 mmol, 180 mg) in EtOH:H_2_O (1:10), three mmol of I_2_ were added and the mixture was stirred for 24 h at 25 °C. Afterwards, 15 mL of CH_2_Cl_2_ were added and treated with Na_2_S_2_O_3_ 20% solution. The organic layer was separated, dried over anhydrous Na_2_SO_4_ and evaporated under reduced pressure. The crude product was then adsorbed on basic alumina (Brockmann I) and submitted to heating at 150 °C for 30 min. After extraction with CH_2_Cl_2_ and evaporation of the solvent under reduced pressure, the crude product was purified on a silica gel column using *n*-hexane:CH_2_Cl_2_ (1:1) as eluent to give 7% yield of **1e** as a white amorphous solid.

*7-methoxy-5-[(2 S,3 S)-7-methoxy-3-methyl-5-[(E)-prop-1-enyl]-2,3-dihydrobenzofuran-2-yl]-2-methyl-benzofuran (****1e****)*. ^1^H NMR (CDCl_3_) δ 7.21 (br s, H-2′), 7.19 (br s, H-6′), 7.05 (br s, H-2 and H-α), 6.83 (s, H-6), 6.49–6.44 (m, H-7′), 6.25–6.20 (m, H-9′), 6.06–6.03 (m, H-8′), 5.11 (d, *J* = 9.4 Hz, H-7), 4.03 (s, OCH_3_), 3.98 (s, OCH_3_), 3.46 (dq, *J* = 13.6 and 6.8 Hz, H-8), 2.41 (s, H-γ), 1.38 (d, *J* = 6.8 Hz, H-9). ^13^C NMR (CDCl_3_) δ 149.6 (C-5′), 148.1 (C-4′), 146.6 (C-6′), 145.4 (C-7′), 144.1 (C-3), 142.0 (C-β), 133.3 (C-4), 133.2 (C-2), 133.0 (C-6), 132.3 (C-2′), 123.5 (C-8′), 119.1 (C-1), 115.7 (C-9′), 113.1 (C-3′), 110.1 (C-α), 109.9 (C-1′), 109.2 (C-5), 93.6 (C-7), 55.9 (OCH_3_), 45.6 (C-8), 17.7 (C-9), 9.6 (C-γ). HRESIMS *m/z* 365.1750 [M + H]^+^ (calcd. for C_23_H_25_O_4_ 365.1753).

### Log P estimation

Log P values were calculated *in silico* using the Marvin Sketch 17.28.0 software (Chemaxon, Inc.). The software employs a weighted method comprised by the PhysProp data mixed with the Klopman’s and Viswanadhan’s calculation methods. The parameters for calculation were the default definition from the software (electrolyte concentrations for Na^+^, K^+^ and Cl^−^ = 0.1 mol/L). The calculated values (Clog P) are presented in Table 1.

### Bioassays procedures

#### Experimental animals

The animal breeding facility of the Adolfo Lutz Institute (São Paulo, Brazil) provided the animal models (BALB/c mice) used in this study. Male BALB/c mice received water and food ad libitum and were maintained in sterilized cages in a controlled environment. All experimental procedures were approved by the Animal Care and Use Committee from *Instituto Adolfo Lutz* – Secretary of Health of São Paulo State - Brazil (Project number 04/2016), in accordance with the Guide for the Care and Use of Laboratory Animals from the National Academy of Sciences.

#### Trypomastigotes and mammalian cell lines maintenance

Rhesus monkey kidney cells (LLC-MK2-ATCC CCL 7) were used for the maintenance of trypomastigotes of *T. cruzi* (Y strain) using RPMI-1640 medium supplemented with 2% fetal bovine serum (FBS). The cells and parasites were kept at 37 °C in a humidified atmosphere containing 5% CO_2_. Peritoneal macrophages, used in the experiments of anti-amastigote assay, were obtained by washing the peritoneal cavity of BALB/c mice, with RPMI-1640 medium supplemented with 10% FBS and kept at 37 °C in a 5% CO_2_ humidified incubator. Murine conjunctive cells (NCTC clone 929, ATCC) and LLC-MK2 were kept in RPMI-1640 supplemented with 10% FBS at the same conditions described above.

#### Anti-trypomastigote assay

To obtain 50% inhibitory concentration (IC_50_) values against *T. cruzi*, free trypomastigotes-LLC-MK2 derived, were counted in a Neubauer hemocytometer, seeded at 1 × 10^6^ cells/well (96-well plates) and incubated with serial dilutions of tested compounds (150–1.71 µM), during 24 h in RPMI-1640 medium at 37 °C in a 5% CO_2_ humidified incubator. After, resazurin (0.011% in PBS) was added to check the viability of the parasites by 24 hours at 37 °C in a 5% CO_2_ humidified incubator. Benznidazole was used as the standard drug. The optical density was determined in FilterMax F5 (Molecular Devices) at 570 nm^38^.

#### Anti-amastigote assay

To obtain 50% inhibitory concentration (IC_50_) values against intracellular amastigotes, peritoneal macrophages collected from the peritoneal cavity of BALB/c mice were used. The cells were plated on a 16-well chamber slide – NUNC (Thermo Fisher Scientific) at 1 × 10^5^ cells/well and incubated for 24 h at 37 °C in a 5% CO_2_ humidified incubator. Next, free trypomastigotes-LLC-MK2 derived, were washed in RPMI-1640 medium, counted and used to infect the macrophages previously plated (10:1, parasite: macrophage ratio). After an incubation of 2 h at 37 °C (5% CO_2_ humidified incubator), residual free parasites were removed by washing with RPMI-1640 medium. Tested compounds were subsequently incubated with infected macrophages for 48 h at 37 °C (5% CO_2_ humidified incubator) in different nontoxic concentrations. Benznidazole was used as standard drug. At the end of the assay, slides were fixed with MeOH and stained with Giemsa, counted under a light microscope (EVOS M5000, Thermo, USA) and IC_50_ values were determined by the infection index^39^.

#### Cytotoxicity against mammalian cells

NCTC cells-clone L929 (6 × 10^4^ cells/well) were seeded and incubated with tested compounds (200–1.56 µM) for 48 h at 37 °C in a 5% CO_2_ humidified incubator. Cytotoxic concentration (CC_50_) was determined by MTT assay^40^. Optical density was determined in FilterMax F5 (Molecular Devices) at 570 nm. Selectivity Index (SI) was determined using the following ratio: CC_50_ against NCTC cells/IC_50_ against parasites.

#### Statistical analysis

IC_50_ and CC_50_ values were calculated using a sigmoid dose-response curves in Graph–Pad Prism 5.0 software (GraphPad Software - San Diego, CA, USA). For the mechanism of action studies one-way ANOVA (Turkey’s Multiple Comparison test) was used for significance (p < 0.05). The assays were repeated at least twice and the samples were tested in duplicate.

#### Assessment of the apparent permeability through PAMPA intestinal model

Intestinal permeability of tested compounds was estimated applying the PAMPA model previously developed and validated^37^. Briefly, stock solutions were prepared in dimethyl sulfoxide (DMSO) at the concentrations of 10 mM and then diluted with Tris buffer to give the final concentration donor solution at 300 µM and 5% DMSO. The assay procedure was initiated by filling each well of the microtiter plate (MultiScreen, catalogue no. MATRNPS50, Millipore Corporation, Bedford, MA, USA) with 300 μL of each donor drug solution. Carefully, and avoiding the pipette tip contact with the filter, the hydrophobic filter (0.45 μm) of each acceptor well of the 96-well microfilter plate (MultiScreen-IP, catalogue no. MAIPNTR10, Millipore Corporation, Bedford, MA, USA) was adsorbed with 6 μL of the artificial lipid solution which was composed of 2% of L-α-phosphatidylcholine from soybean dissolved, by sonication, in *n*-dodecane. Immediately after this application, 150 μL of Tris buffer containing 5% DMSO was added to the receiving well. The receiving well was mounted on the donor plate, keeping the underside of the membrane in contact with the donor solution. The assembled donor–acceptor plates were incubated under constant stirring (3 g) at 25 °C for approximately 16 h. Subsequently, the quantity of each compound presented at the receptor solution (150 μL) was determined by UV/VIS spectrophotometrically. The experiments were performed in hexaplicates (n = 6) and the apparent permeability coefficient (Papp) calculated in centimeters per second (cm/s), together with the standard deviation (SD). Compounds with Papp equal to or higher than 1.0 × 10^–6^ cm/s are classified as with high intestinal absorption (>85%) but if it is higher than 1.0 × 10^−5^ cm/s they also exhibit a plasma protein binding higher than 90%^41^.

#### Assessment of the apparent permeability through PAMPA-BBB model

The methodology herein applied was similar to that described in the previous section, however with the purpose of assessing the permeability of the compounds through the blood brain barrier (BBB). Thus, stock solutions (10 mM) of each test compound were prepared in DMSO and diluted with phosphate buffered saline (PBS) at pH 7.4. The final concentration of donor solutions was 300 μM and DMSO of 5%. Artificial membrane lipid solutions were prepared daily by dissolving, in *n*-dodecane, the porcine brain lipid extracted as described^40^ at the final concentration of 2% (m/v). PAMPA procedure was similar to that described in the previous section although, in PAMPA-BBB, the donor and acceptor solutions were prepared using phosphate buffer saline (PBS) at pH 7.4 and the Papp was obtained through the equation previously reported^42^. Accordingly, compounds with values of Papp equal to or higher than 2.0 cm/s are classified as permeable through BBB while those with compounds with lower values of Papp are classified as compounds that do not cross BBB.

#### Cell membrane permeability analysis

The action of compounds **1d**, **1e**, **3d** and **4a** in the cell membrane permeability were evaluated in *T. cruzi* trypomastigotes (2 × 10^6^/well) seeded in 96-well black polystyrene microplates. Parasites were washed and incubated in the dark with 1 μM SYTOX Green probe (Molecular Probes) in HANKS’ balanced salts solution (HBSS; Sigma-Aldrich) supplemented with 10 mM D-Glucose (HBSS + Glu) in 96-well black polystyrene microplates^43,44^. Each compound was added (t = 0 min) at IC_50_ concentration and fluorescence levels were measured every 20 min for up to 120 min. The maximum permeabilization was obtained with the addiction of 0.5% Triton X-100. Fluorescence intensities were determined using a fluorimetric microplate reader (FilterMax F5 Multi-Mode Microplate Reader-Molecular Devices) with excitation and emission wavelengths of 485 and 520 nm, respectively. The following internal controls were used in the evaluation: i) the background fluorescence of the compound at the respective wavelengths, ii) the possible interference of DMSO.

#### Analysis of reactive oxygen species (ROS)

Trypomastigotes (2 × 10^6^ parasites/well) were seeded in 96-well black polystyrene microplates and treated with compounds **1d**, **1e**, **3d** and **4a** (at IC_50_ value) for 1 and 2 h in HBSS + Glu at 37 °C. After, H_2_DCFDA probe (Molecular Probes) was added (5 µM) and incubated by 15 min^44^. The fluorescence intensity was measure using a fluorimetric microplate reader (FilterMax F5 Multi-Mode, Molecular Devices) with excitation and emission wavelengths of 485 and 520 nm, respectively. Azide (10 mM) was used as positive control and untreated parasites were used as negative control.

#### Analysis of ATP generation

Trypomastigotes (2 × 10^6^ parasites/well) were treated with compounds **1d**, **1e**, **3d** and **4a** (at IC_50_ value) in HBSS + Glu for 1 and 2 h at 37 °C. Untreated parasites and treated with the mitochondrial uncoupler CCCP (carbonyl cyanide *m*-chlorophenylhydrazone - Sigma) at 100 µM, were used as positive and negative controls, respectively. The trypomastigotes were lysed using 0.5% Triton X-100 and mixed with a standard reaction buffer (ATP Determination Kit, Molecular Probes) containing DTT (1 mM), luciferin (0.5 mM) and firefly luciferase (1.25 µg/mL). Luminescence intensity was measured using a luminometer (FilterMax F5 Multi-Mode, Molecular Devices) and the amount of ATP was calculated from an ATP standard curve^45^.

#### Evaluation of the mitochondrial membrane potential (ΔΨm)

The ΔΨm were analyzed by flow cytometry (Attune NxT flow cytometer - ThermoFisher) with the probe 5,5′,6,6′-tetrachloro-1,1′,3,3′-tetraethylbenzimidazole carbocyanide iodide (JC-1, ThermoFisher). The ratio between red/green fluorescence intensities (BL-2/BL-1; 590 nm/530 nm) were calculated^45,46^. Trypomastigotes (2 × 10^6^/tube) treated for 2 h with the selected compounds (IC_50_ values) were washed in HBSS + Glu and ressuspended with JC-1 dye at a final concentration of 10 μM. The parasites were incubated in the dark for 10 min at 37 °C and washed in HBSS + Glu to eliminate the non-internalized dye. As internal controls of the assay were used: (i) non-treated cells and (ii) trypomastigotes treated with 100 μM CCCP.

## Supplementary information

Supplementary Information.
