# Supplementary material for: Improving the drug-likeness of inspiring natural products - evaluation of the antiparasitic activity against Trypanosoma cruzi through semi-synthetic and simplified analogues of licarin A
Source: Sci Rep. 2020 Mar 25;10:5467. doi: 10.1038/s41598-020-62352-w (PMC7096397; doi:10.1038/s41598-020-62352-w)
Supplement: Supplementary file 1 — Supplementary Information. [file 41598_2020_62352_MOESM1_ESM.docx]

**Improving the drug-likeness of inspiring natural products - evaluation of the antiparasitic activity against *Trypanosoma cruzi* through semi-synthetic and simplified analogues of licarin A**

Thiago R. Morais^1^, Geanne A. Alves Conserva^2^, Marina T. Varela^1^,

Thais A. Costa-Silva^2^, Fernanda Thevenard^2^, Vitor Ponci^1^, Ana Fortuna^3,4^,

Amílcar C. Falcão^3,4^, Andre G. Tempone^5^, João Paulo S. Fernandes^1,^* &

João Henrique G. Lago^2,^*

^1^Institute of Environmental, Chemical and Pharmaceutical Sciences, Universidade Federal de São Paulo, São Paulo, 09972-270, Brazil.

^2^Center of Natural Sciences and Humanities, Universidade Federal do ABC, São Paulo, 09210-580, Brazil.

^3^Laboratory of Pharmacology, Faculty of Pharmacy of University of Coimbra, 3000-370, Coimbra, Portugal.

^4^CIBIT/ICNAS – Coimbra Institute for Biomedical Imaging and Translational Research, University of Coimbra, 3000-370, Coimbra, Portugal.

^5^Centre for Parasitology and Mycology, Instituto Adolfo Lutz, São Paulo, 01246-000, Brazil.

**SUPPORTING INFORMATION**

NMR and MS data for compounds **1** and **1a – 1e**


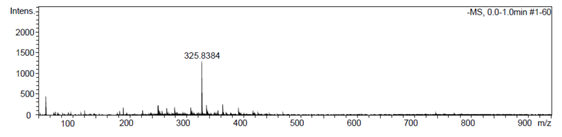


**Figure 1**. HRESIMS spectrum (negative mode) of compound **1**


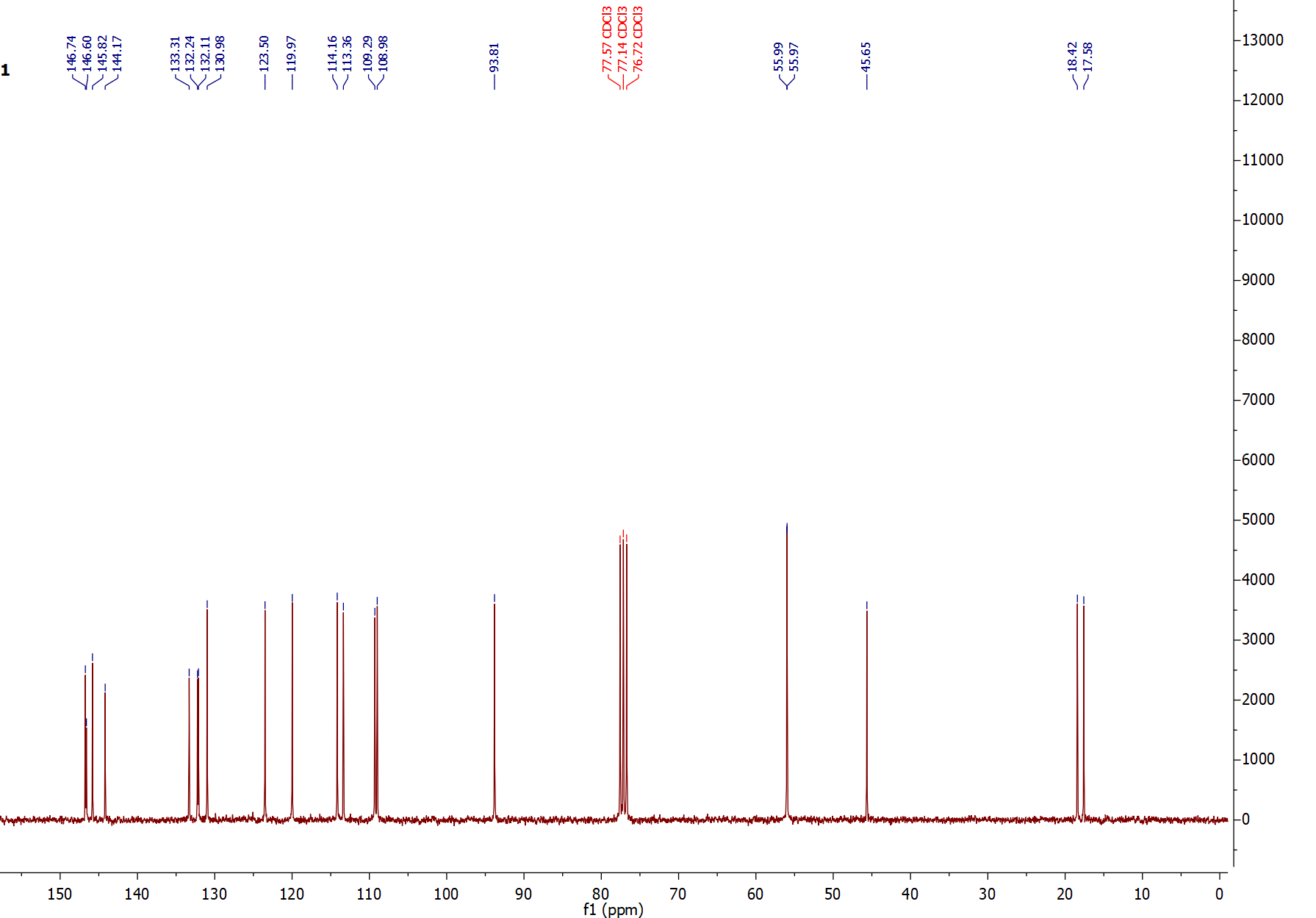


**Figure 2**. ^13^C NMR spectrum of compound **1** (δ/ppm, 75 MHz, CDCl_3_)


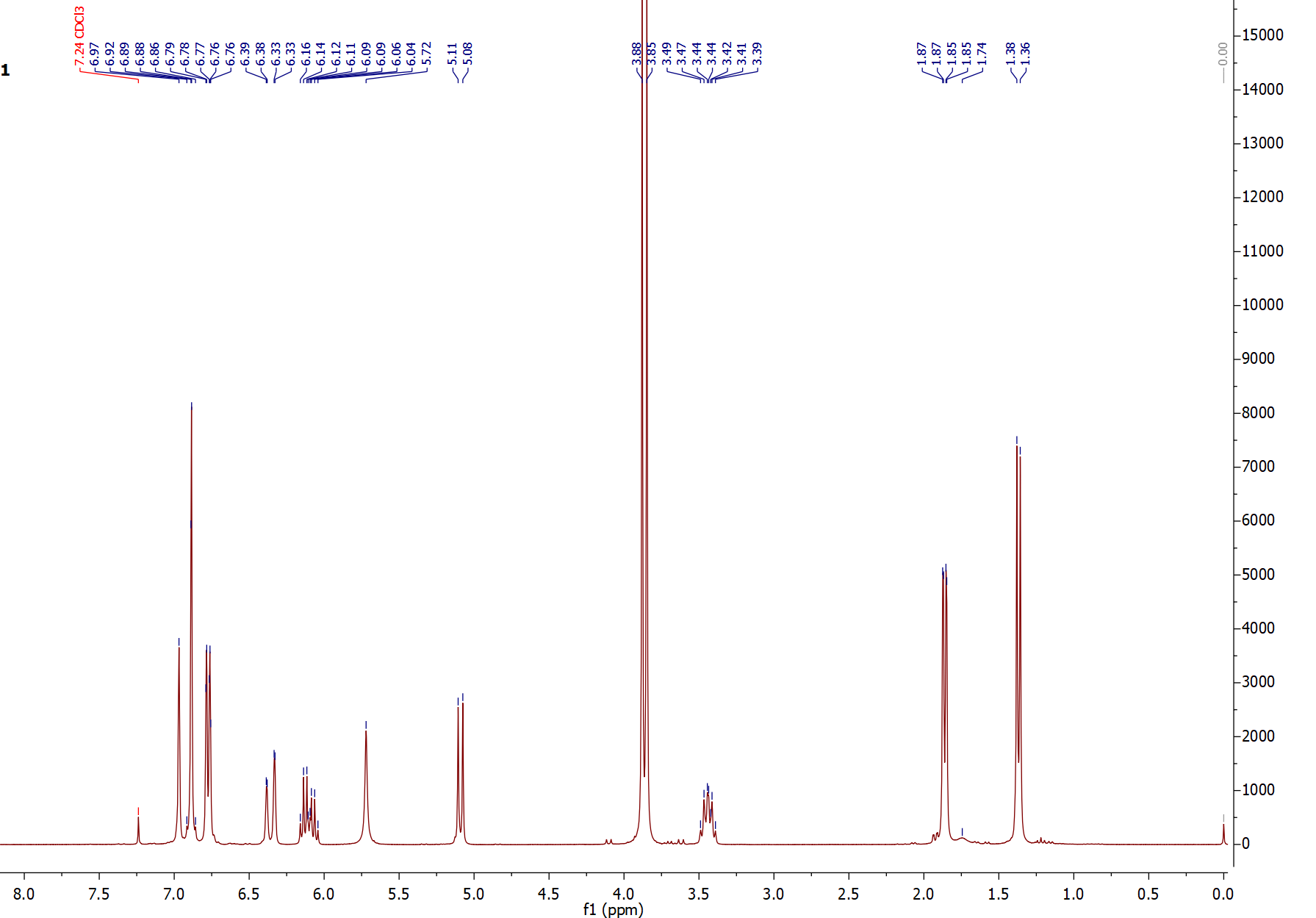


**Figure 3**. ^1^H NMR spectrum of compound **1** (δ/ppm, 300 MHz, CDCl_3_)

**
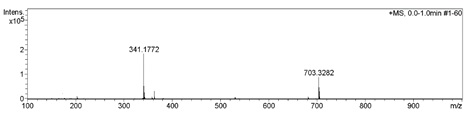
**

**Figure 4**. HRESIMS spectrum (positive mode) of compound **1a**


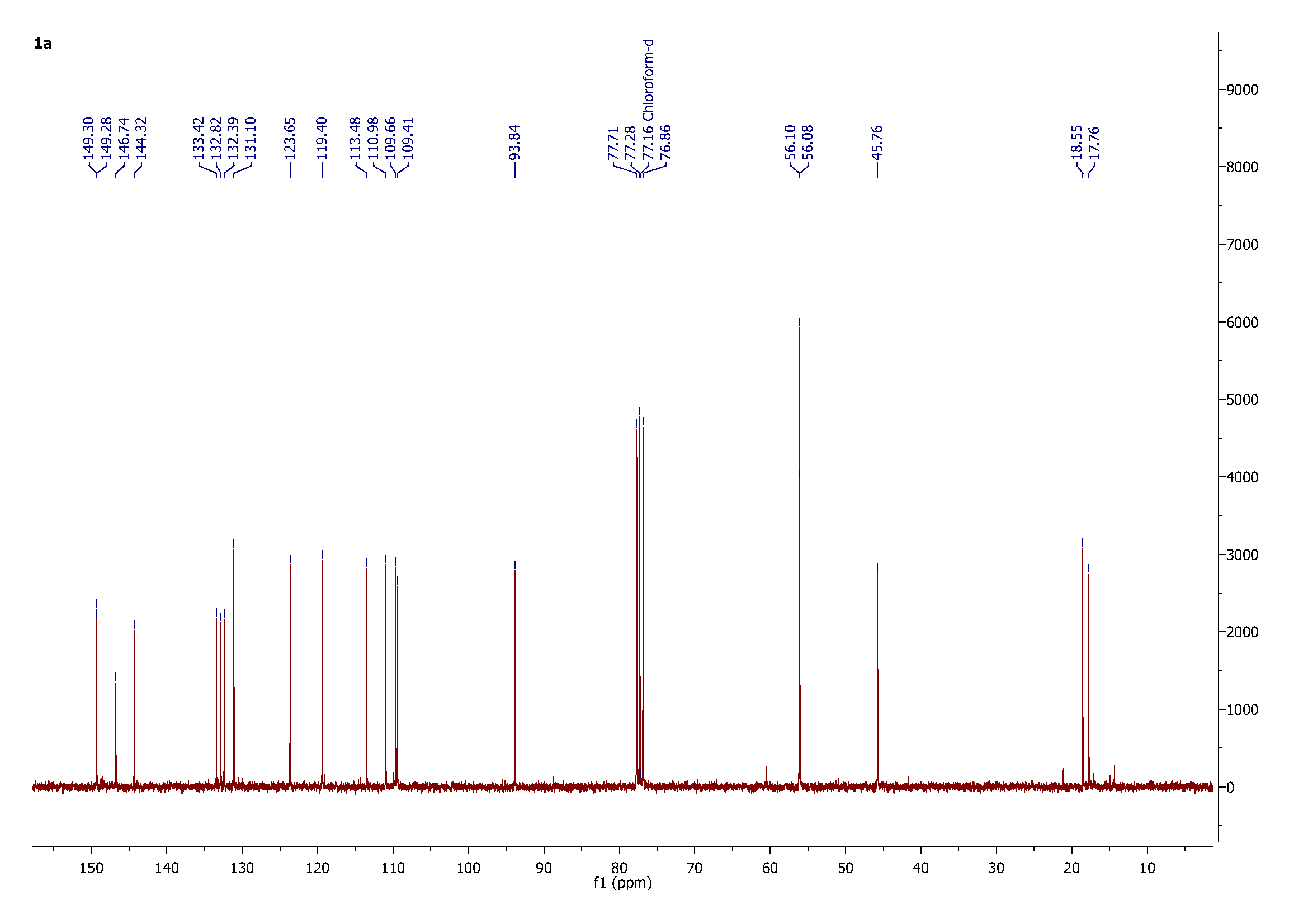
 **Figure 5**. ^13^C NMR spectrum of compound **1a** (δ/ppm, 75 MHz, CDCl_3_)


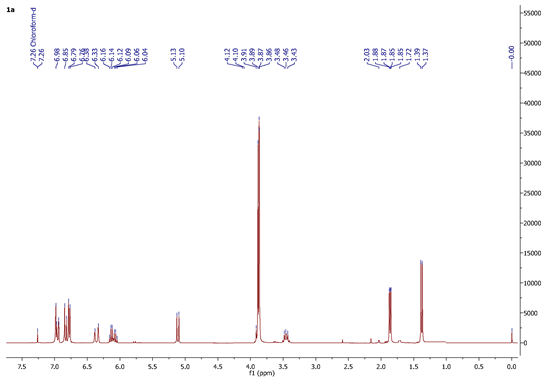


**Figure 6**. ^1^H NMR spectrum of compound **1a** (δ/ppm, 300 MHz, CDCl_3_)

**
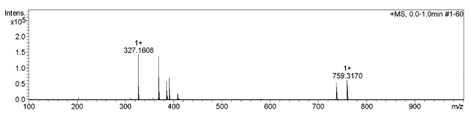
**

**Figure 7**. HRESIMS spectrum (positive mode) of compound **1b**

**
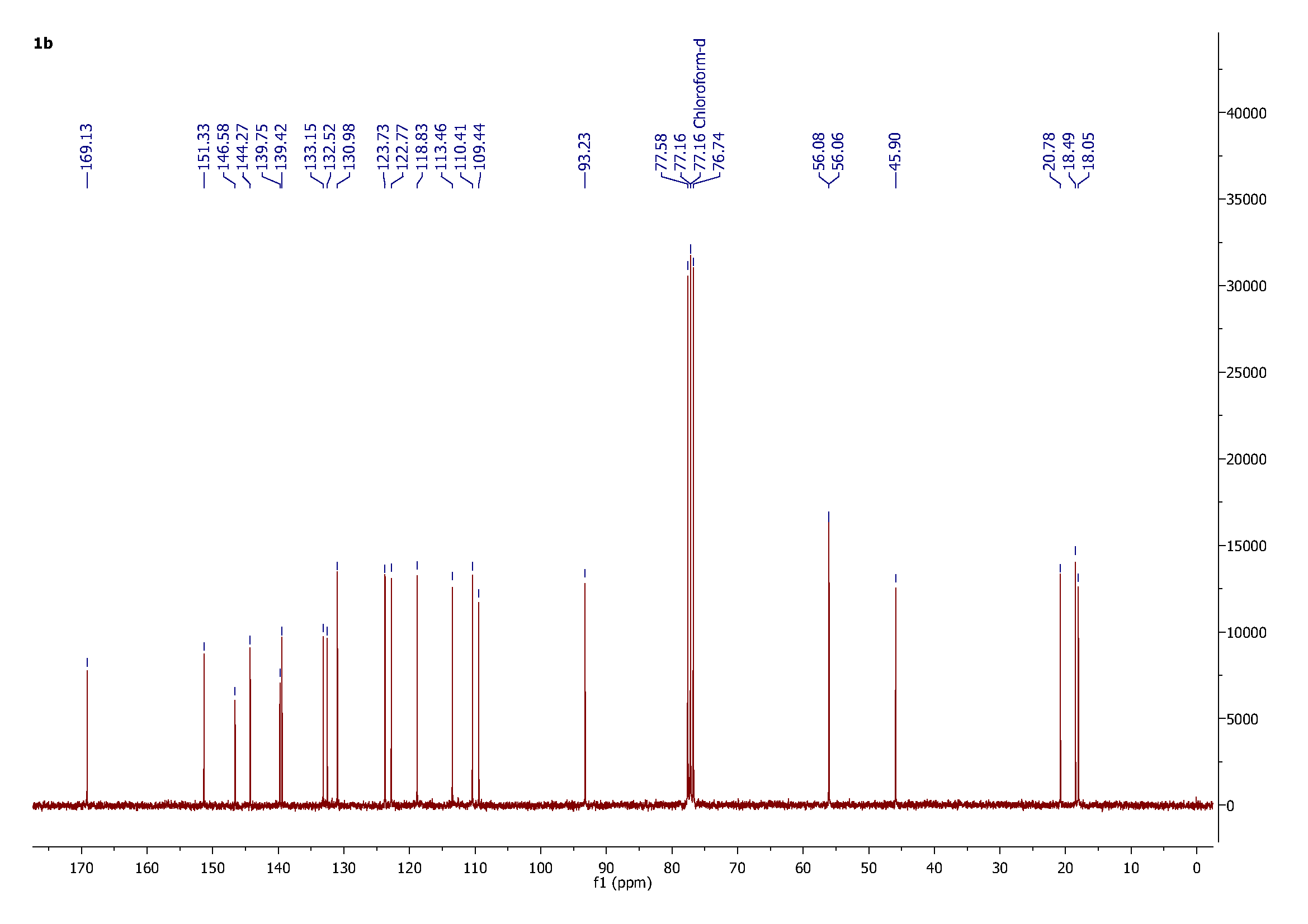
Figure 8**. ^13^C NMR spectrum of compound **1b** (δ/ppm, 75 MHz, CDCl_3_)

**
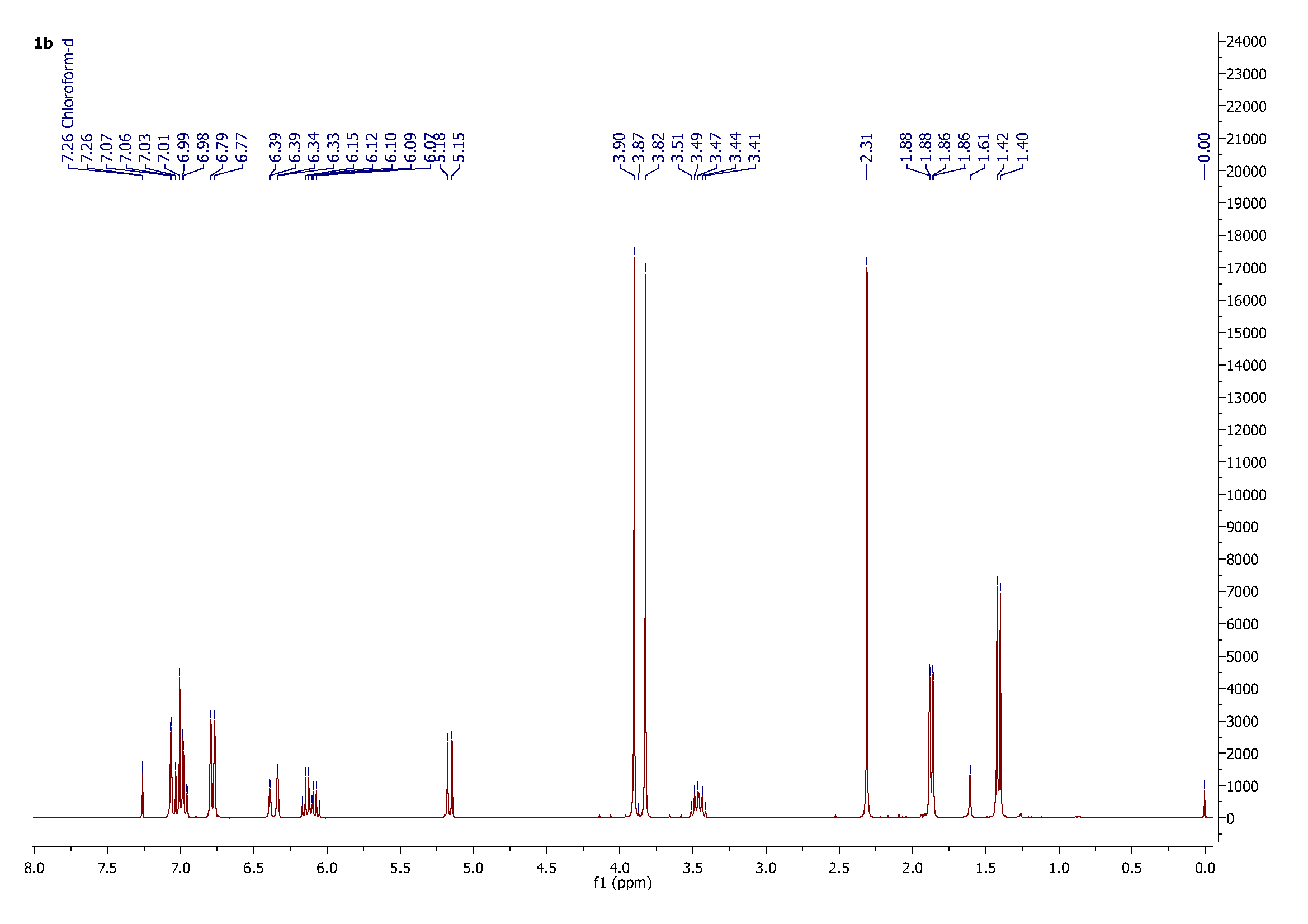
**

**Figure 9**. ^1^H NMR spectrum of compound **1b** (δ/ppm, 300 MHz, CDCl_3_)

**
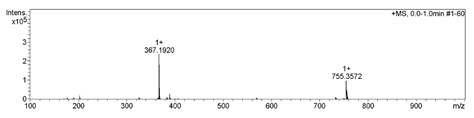
**

**Figure 10**. HRESIMS spectrum (positive mode) of compound **1c**

**
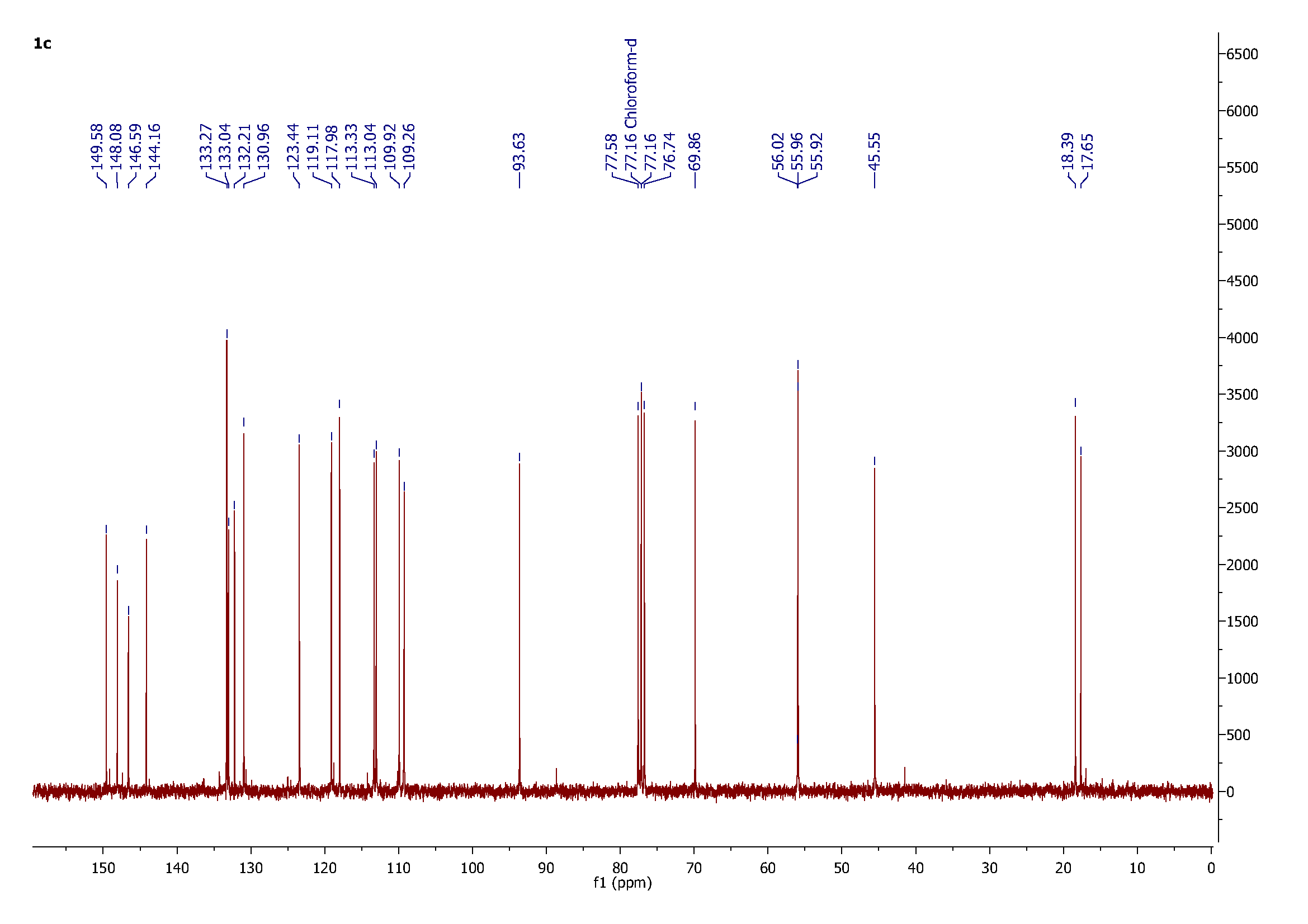
 Figure 11**. ^13^C NMR spectrum of compound **1c** (δ/ppm, 75 MHz, CDCl_3_)

**
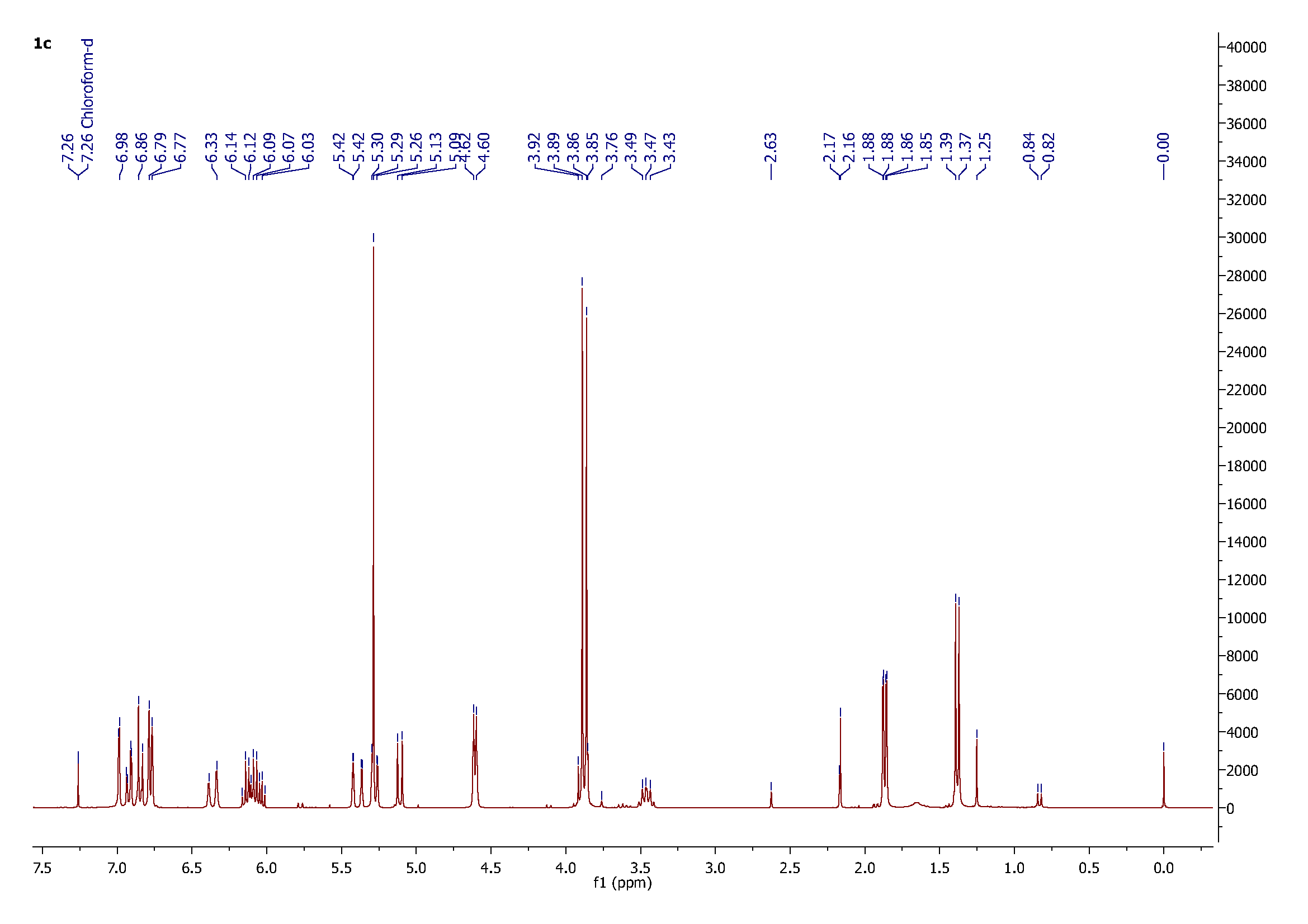
 Figure 12**. ^1^H NMR spectrum of compound **1c** (δ/ppm, 300 MHz, CDCl_3_)

**
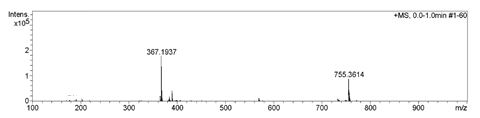
**

**Figure 13**. HRESIMS spectrum (negative mode) of compound **1d**


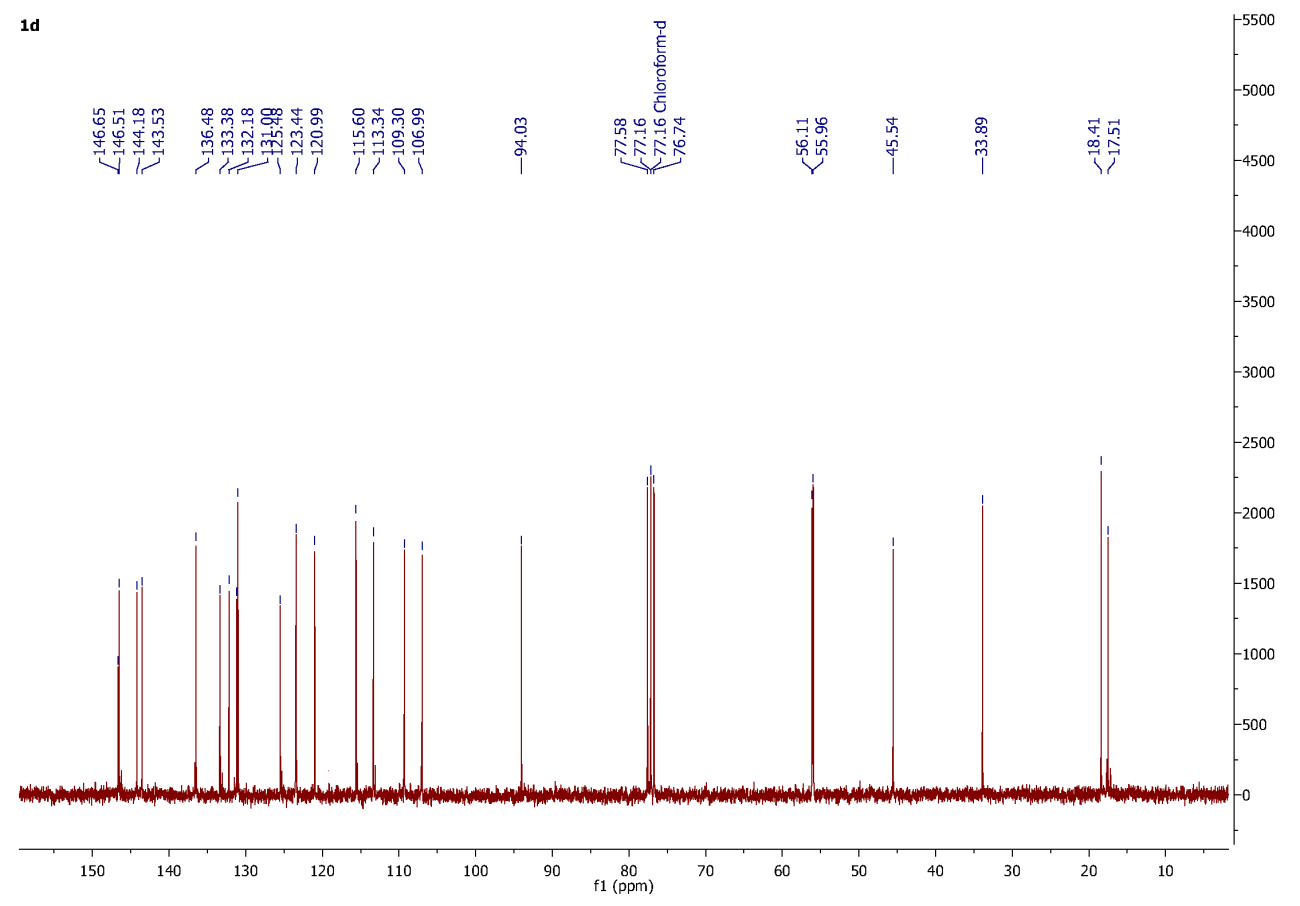


**Figure 14**. ^13^C NMR spectrum of compound **1d** (δ/ppm, 75 MHz, CDCl_3_)


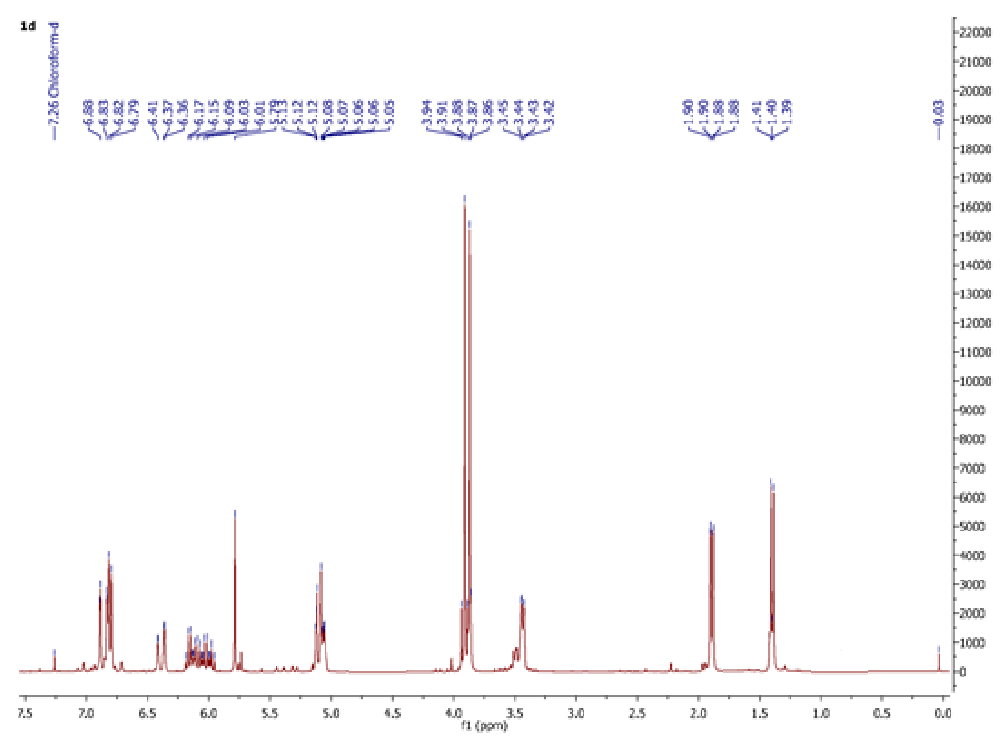


**Figure 15**. ^1^H NMR spectrum of compound **1d** (δ/ppm, 300 MHz, CDCl_3_)


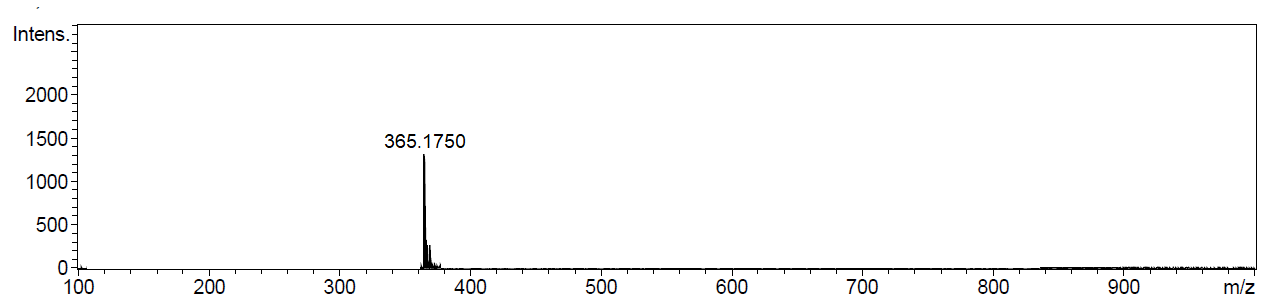


**Figure 16**. HRESIMS spectrum (positive mode) of compound **1e**


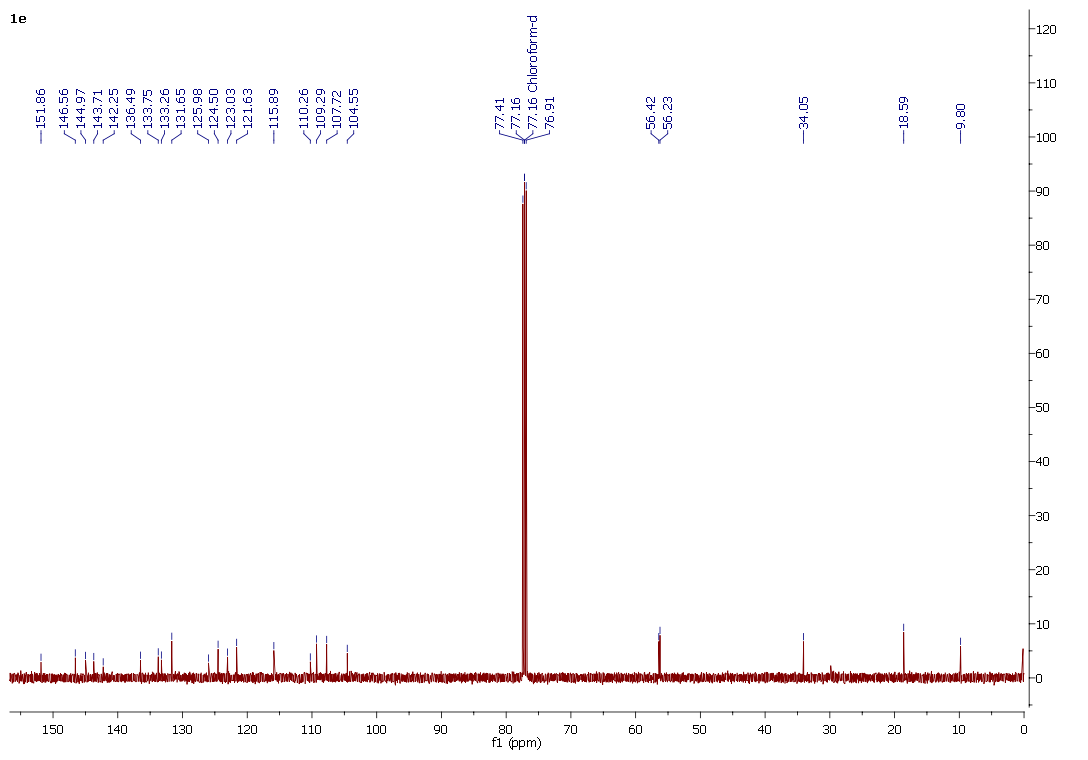
 **Figure 17**. ^13^C NMR spectrum of compound **1e** (δ/ppm, 75 MHz, CDCl_3_)


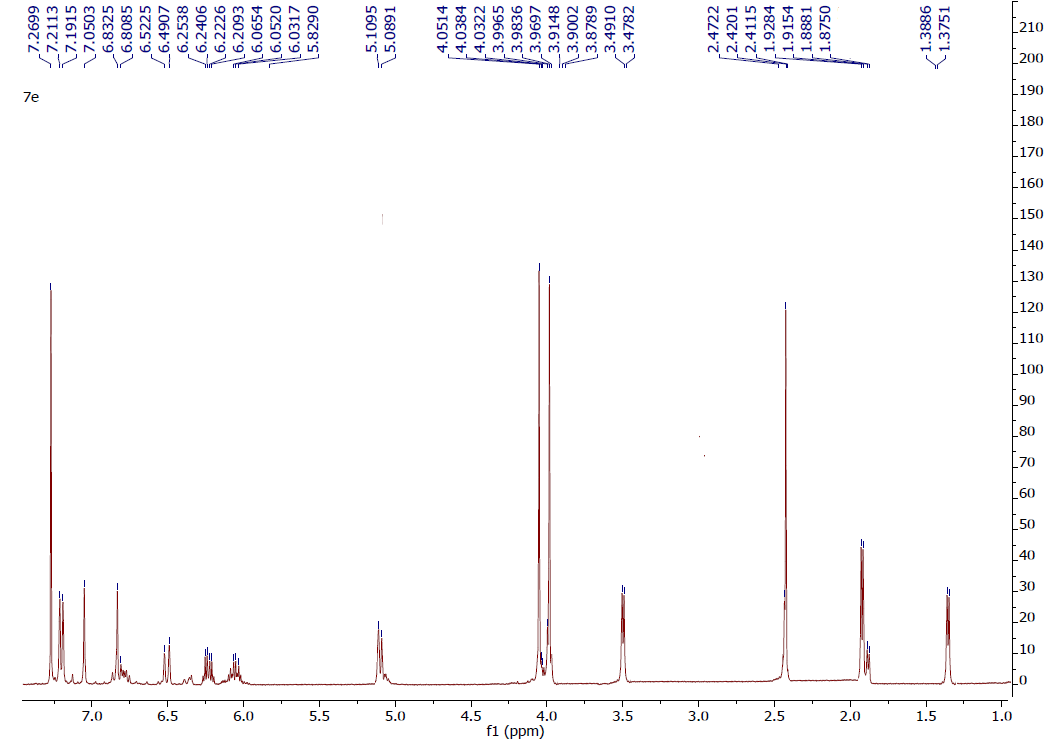


**Figure 18**. ^1^H NMR spectrum of compound **1e** (δ/ppm, 300 MHz, CDCl_3_)
